# New Fossil Lepidoptera (Insecta: Amphiesmenoptera) from the Middle Jurassic Jiulongshan Formation of Northeastern China

**DOI:** 10.1371/journal.pone.0079500

**Published:** 2013-11-22

**Authors:** Weiting Zhang, Chungkun Shih, Conrad C. Labandeira, Jae-Cheon Sohn, Donald R. Davis, Jorge A. Santiago-Blay, Oliver Flint, Dong Ren

**Affiliations:** 1 College of Life Sciences, Capital Normal University, Beijing, China; 2 Department of Paleobiology, National Museum of Natural History, Smithsonian Institution, Washington, Distirict of Columbia, United States of America; 3 Department of Entomology and BEES Program, University of Maryland, College Park, Maryland, United States of America; 4 Department of Entomology, National Museum of Natural History, Smithsonian Institution, Washington, District of Columbia, United States of America; 5 Department of Crop and Agroenvironmental Sciences, University of Puerto Rico Mayagüez, Puerto Rico, United States of America; 6 Geoscience Museum, Shijiazhuang University of Economics, Shijiazhuang, China; University of Kansas, United States of America

## Abstract

**Background:**

The early history of the Lepidoptera is poorly known, a feature attributable to an inadequate preservational potential and an exceptionally low occurrence of moth fossils in relevant mid-Mesozoic deposits. In this study, we examine a particularly rich assemblage of morphologically basal moths that contribute significantly toward the understanding of early lepidopteran biodiversity.

**Methodology/Principal Findings:**

Our documentation of early fossil moths involved light- and scanning electron microscopic examination of specimens, supported by various illumination and specimen contrast techniques. A total of 20 moths were collected from the late Middle Jurassic Jiulongshan Formation in Northeastern China. Our principal results were the recognition and description of seven new genera and seven new species assigned to the Eolepidopterigidae; one new genus with four new species assigned to the Mesokristenseniidae; three new genera with three new species assigned to the Ascololepidopterigidae fam. nov.; and one specimen unassigned to family. Lepidopteran assignment of these taxa is supported by apomorphies of extant lineages, including the M_1_ vein, after separation from the M_2_ vein, subtending an angle greater than 60 degrees that is sharply angulate at the junction with the r–m crossvein (variable in Trichoptera); presence of a foretibial epiphysis; the forewing M vein often bearing three branches; and the presence of piliform scales along wing veins.

**Conclusions/Significance:**

The diversity of these late Middle Jurassic lepidopterans supports a conclusion that the Lepidoptera–Trichoptera divergence occurred by the Early Jurassic.

## Introduction

**Figure 1:**
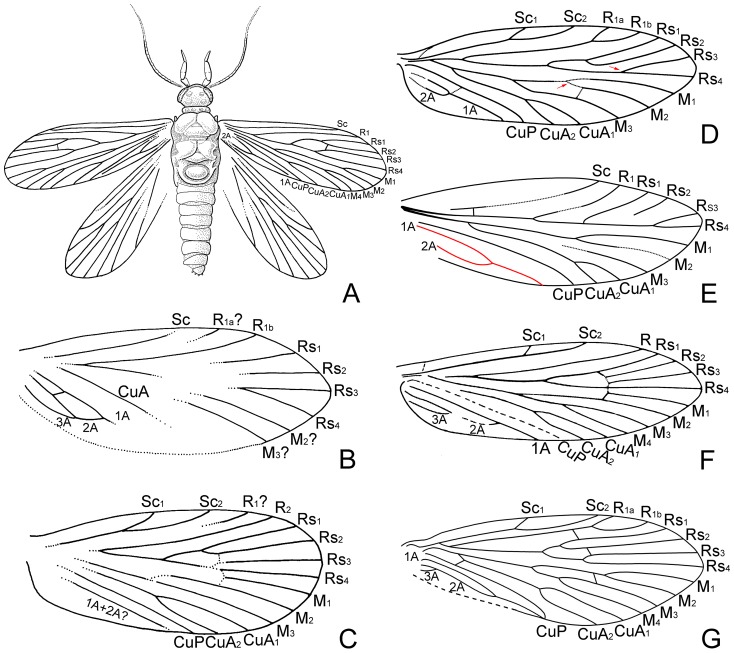
A stem-group amphiesmenopteran, lepidopterans, and a trichopteran. (A), A stem-group amphiesmenopteran, *Necrotaulius tener*, modified after Grimaldi and Engel [5]. (B-F), Lepidopteran forewings. (B), *Archaeolepis mane*, from Grimaldi and Engel [5]. (C), Forewing of an Early Jurassic Germany lepidopteran, from Grimaldi and Engel [5]. (D), *Undopterix sukatshevae*, modified after Skalski [10]; the red arrowhead showing Rs_3+4_ furcation beyond the M_1+2_ furcation. (E), *Netoxena nana*, modified after Martins-Neto [24]; red line indicates a single-Y configuration. (F), *Mesokristensenia sinica*, modified after Huang *et al*. [25]. (G), A trichopteran, *Juraphilopotamus lubricus*, modified after Gao *et al*. [43].

The Lepidoptera, or butterflies and moths, are one of the most speciose lineages of herbivores, currently including about 157,500 described species encompassing the four suborders of Zeugloptera, Aglossata, Heterobathmiina, and Glossata [1]. In contrast to the documented paleodiversity of otherdiverse insect lineages [2], the Lepidopteran fossil record is depauperate [3]. Only two extant suborders, Zeugloptera and Glossata, and an extinct suborder, Eolepidopterigina proposed by Rasnitsyn [4], are represented in the fossil record. Speciosity of the basal lepidopteran lineages is minimal in the fossil record, as in the extant fauna. This may be a consequence of the Lepidoptera once constituting a minor group of the world’s fauna, following their evolution from the stem-group, the Amphiesmenoptera (Fig. 1A) [5], a major holometabolan lineage comprising two distinctive insect orders, the Lepidoptera and the Trichoptera. The taxonomic boundary between Lepidoptera and Trichoptera is often obscured in their early fossil record [3], attributable to an overall morphological similarity and presence of few apomorphic characters visible in fossil specimens [5].

Because of the rarity of Mesozoic Lepidoptera, only 24 genera, of which two are extant, have been described from the Mesozoic (Table 1) [3], [6]–[8]. In addition, undescribed Mesozoic fossils have been reported, including unequivocal Lepidoptera larvae [5], [9], and other evidence such as unequivocal or possible Lepidoptera wing scales [10]–[12] and unequivocal Lepidoptera leaf mines [13], [14]. Mesozoic fossil Lepidoptera are principally assigned to two extinct families, the Eolepidopterigidae and the Mesokristenseniidae, and an extant family, the Micropterigidae, the latter considered the most basal extant lineage of Lepidoptera [15], [16]. In addition, Kozlov erected an extinct family, the Undopterigidae to accommodate *Undopterix* Skalski, 1979 [10], but its phylogenetic placement is uncertain.

**Table 1:** Known fossil records of Mesozoic Lepidoptera.

Suborder	Family	Genus	Species	Epoch	Country	Reference
?	Archaeolepidae	*Archaeolepis* Whalley, 1985	*A. mane* Whalley, 1985	J1	United Kingdom	[18]
Eolepidopterigina	Eolepidopterigidae	*Eolepidopterix* Rasnitsyn, 1983	*E. jurassica* Rasnitsyn, 1983	J3	Kazakhstan	[4]
Eolepidopterigina	Eolepidopterigidae	*Palaeolepidopterix* Kozlov, 1989	*P. aurea* Kozlov, 1989	J3	Kazakhstan	[40]
Eolepidopterigina	Eolepidopterigidae	*Daiopterix* Skalski, 1984	*D. rasnitsyni* Skalski, 1984	K1	Russia	[23]
Eolepidopterigina	Eolepidopterigidae	*Daiopterix* Skalski, 1984	*D. olgae* Kozlov, 1989	K1	Russia	[40]
Eolepidopterigina	Eolepidopterigidae	*Netoxena* Sohn, 2012	*N. nana* (Martins-Neto, 1999)	K1	Brazil	[24], [52]
Eolepidopterigina	Eolepidopterigidae	*Undopterix* Skalski, 1979	*U. sukatshevae* Skalski, 1979	K1	Russia	[10]
Eolepidopterigina	Eolepidopterigidae	*Undopterix* Skalski, 1979	*U. caririensis* Martins-Neto & Vulcano,1989	K1	Brazil	[39]
Eolepidopterigina	Eolepidopterigidae	*Gracileopterix* Neto & Vulcano,1989	*G. pulchra* Martins-Neto & Vulcano, 1989	K1	Brazil	[39]
Zeugloptera	Micropterigidae	*Sabatinca* Walker, 1863	*S. perveta* (Cockerell, 1919)	K1	Burma	[12], [42]
Zeugloptera	Micropterigidae	*Parasabatinca* Whalley, 1978	*P. aftimacrai* Whalley, 1978	K1	Lebanon	[45]
Zeugloptera	Micropterigidae	*Parasabatinca* Whalley, 1978	*P. caldasae* Martins-Neto & Vulcano, 1989	K1	Brazil	[39]
Zeugloptera	Micropterigidae	*Palaeosabatinca* Kozlov, 1988	*P. zherichini* Kozlov, 1988	K1	Russia	[16]
Zeugloptera	Micropterigidae	?	?	J1	Germany	[21], [22]
Zeugloptera	Micropterigidae	?	?	K1	Lebanon	[53]
Zeugloptera	Micropterigidae	?	?	K1	Burma	[5], [52], [54]
Zeugloptera	Micropterigidae	?	?	K1	Spain	[52], [55]
?	?	?	?	?	Burma	[52], [56]
Zeugloptera	?Micropterigidae	*Auliepterix* Kozlov, 1989	*A. mirabilis* Kozlov, 1989	J3	Kazakhstan	[40]
Zeugloptera	?Micropterigidae	*Auliepterix* Kozlov, 1989	*A. minima* Kozlov, 1989	J3/K1	Mongolia	[40]
Zeugloptera	?Micropterigidae	?	?	J3/K1	France	[9], [16]
Zeugloptera	?Micropterigidae	?	?	J3/K1	France	[57]
?	Mesokristenseniidae	*Mesokristensenia* Huang *et al*.,2010	*M. latipenna* Huang *et al*., 2010	J2	China	[25]
?	Mesokristenseniidae	*Mesokristensenia* Huang *et al*.,2010	*M. sinica* Huang *et al*., 2010	J2	China	[25]
?	Mesokristenseniidae	*Mesokristensenia* Huang *et al*.,2010	*M. angustipenna* Huang *et al*., 2010	J2	China	[25]
Glossata	Bucculatricidae	*Bucculatrix* Zeller, 1839	*B. platani* Kozlov, 1988	K2	Kazakhstan	[16]
Glossata	Incurvariidae	?	?	K2	Russia	[10]
Glossata	Incurvariidae	?	?	K1	Brazil	[5]
Glossata	Incurvariidae	?	?	K1	Lebanon	[45]
Glossata	?Mnesarchaeidae	?	?	K2	Russia	[5], [58]
Glossata	?Lophocoronidae	?	?	K2	Russia	[10]
Glossata	?	*Protolepis* Kozlov, 1989	*P. cuprealata* Kozlov, 1989	J3	Kazakhstan	[40]
Glossata	?	*Karataunia* Kozlov, 1989	*K. lapidaria* Kozlov, 1989	J3	Kazakhstan	[40]
?	?	*Archiptilia* Handlirsch, 1939	*A. ovata* Handlirsch, 1939	J1	Germany	[21], [59]
?	?	*Epididontus* Handlirsch, 1939	*E. geinitzianus* Handlirsch, 1939	J1	Germany	[21], [59]
?	?	*Metarchitaulius* Handlirsch, 1939	*M. longus* Handlirsch, 1939	J1	Germany	[21], [59]
?	?	*Nannotrichopteron* Handlirsch,1906	*N. gracile* Handlirsch, 1906	J1	Germany	[20], [21]
?	?	*Palaeotaulius* Handlirsch, 1939	*P. vicinus* Handlirsch, 1939	J1	Germany	[21], [59]
?	?	*Pararchitaulius* Handlirsch, 1939	*P. ovalis* Handlirsch, 1939	J1	Germany	[21], [59]
?	?	*Parataulius* Handlirsch, 1939	*P. jurassicus* Handlirsch, 1939	J1	Germany	[21], [59]
?	?	*Pseudorthophlebia* Handlirsch, 1906	*P. platyptera* Handlirsch, 1906	J1	Germany	[20], [21]
?	?	*?Paratrichopteridium* Handlirsch,1906	*P. efossum* Handlirsch, 1939	J1	Germany	[21], [59]
?	?	*?Paratrichopteridium* Handlirsch, 1906	*P. costale* Handlirsch, 1939	J1	Germany	[21], [59]
?	?	*Necrotaulius* Handlirsch, 1906	*N. tener* Sukatsheva, 1990	K1	Russia	[50], [60]
Glossata	?	?	?	K1	Burma	[54]
Glossata	?	?	?	K2	USA	[61]
Glossata^1^	Nepticulidae	?	?	K1	USA	[13]
Glossata^1^	Gracillariidae	?	?	K1	USA	[13]
Glossata (larva)	?	?	?	K1	Lebanon	[62]
Glossata (larva^2^)	?	?	?	K2	Canada	[9]
Glossata (larva)	?	?	?	K1	Lebanon	[5]
?	?	?	?	K1	Spain	[63]

Notes: ^1^Leaf mine;

2 Head capsule. J1, Early Jurassic; J2, Middle Jurassic; J3, Late Jurassic; K1, Early Cretaceous; K2, Late Cretaceous.

The earliest unequivocal lepidopteran fossil hitherto established is *Archaeolepis mane* Whalley, 1985 (Fig. 1B) from the Early Jurassic, about 190 million years ago (Ma), of Dorset, England [5], [18], [19]. The next, more recent, early fossils are eight non-monophyletic genera, dated to approximately 180 Ma, from the uppermost Lias of Dobbertin, Germany [5], which were described by Handlirsch as Necrotauliidae. However, Ansorge [21], [22] disputed the necrotaulid affiliations of the Handlirsch’s specimens and other well-preserved wings from the Grimmen locality of Germany, and assigned most of these to the Lepidoptera on the basis of the 3-branched M vein and the presence of scales on the forewings. These fossils overwhelmingly are represented by wings. A representative forewing is shown in Fig. 1C, indicating a M with 3 branches and the M_1_, separated from the M_2_ at an angle of about 70 degrees, and sharply angulate at the junction with crossvein r–m. Additionally, a forewing of *Undopterix sukatshevae*, within the Undopterigidae [23], is shown in Fig. 1D, as is a forewing of *Netoxena nana* in the Eolepidopterigidae [3], [24], in Fig. 1E. Huang *et al*. [25] described *Mesokristensenia sinica* in the Mesokristenseniidae, the forewing of which is shown in Fig. 1F. For comparison, the forewing of a trichopteran, *Juraphilopotamus lubricus* Wang, Zhao & Ren, 2009 [17], is shown in Fig. 1G.

Recently, we collected 20 well-preserved, nearly complete fossil lepidopterans from the Jiulongshan Formation at Daohugou Village, Shantou Township, Ningcheng County, Inner Mongolia, in China. This deposit was radiometrically dated by ^40^K/^40^Ar at 164–165 Ma [26], a date supported by slightly younger isotopic dates from overlying volcanic deposits [26], [27]. This date corresponds to the Callovian–Oxfordian boundary interval of the latest Middle Jurassic, using the most recent, standard international time scale [28]. The deposit contain beautifully preserved fossils of insects and other animals [29]–[33]. After comparison of these fossils to other Mesozoic lepidopteran specimens, we recognized below eleven new genera and fourteen new species, assigned to the three families, the Eolepidopterigidae, the Mesokristenseniidae, and the Ascololepidopterigidae fam. nov. We used additional taxa to emend diagnoses of the two previously named families, the Eolepidopterigidae and the Mesokristenseniidae. With the fossils reported by Huang *et al*. [25], these new findings, now document the third oldest-known lineage of lepidopterans.

## Materials and Methods

### Light Microscopy, Image Processing and Line Drawings

All specimens were microphotographed and illustrated using a Leica MZ12.5 dissecting microscope accompanied with a drawing tube attachment in Beijing, and an Olympus SZX12 stereomicroscope in Washington, DC. Images were captured using dissecting microscopes equipped with digital cameras and image processing software. The image processing software for the Beijing computer–microscope–camera system was a Nikon DXM 1200C, with NIS-elements D 2.30; and in Washington, DC, an Image-Pro 6.1 (Media Cybernetics) platform. Alcohol was used to increase the contrast of specimen structures for some images. Line drawings in Beijing were made in conjunction with Photoshop CS2 graphic software.

### Electron Microscopy

We used a Philips XL30 environmental scanning electron microscope to examine shrink-wrapped specimens. Compression fossils, such as the material studied herein, generally present minimal topographic relief. This absence of microtopographic differentiation and the uncoated state of the specimens, posed a demand on specimen imaging. By varying the microscopy conditions for each specimen, including sources of illumination, such as back-scattered electrons, gas emission secondary electrons and particle detector adjustment; or alternatively, varying microtopographic aspect, spot size or accelerating voltage; we strove to find the best combination of imaging conditions within a challenging SEM environment.

The fossil specimens studied under vacuum conditions of the electron microscope necessitated a modification of specimen preparation. The presence of a poorly consolidated sedimentary matrix surrounding the fossils resulted in the spalling of particles that interfered with normal operation of the vacuum tube. A protocol was devised that solved this problem. Any matrix material that sloughed from the specimen was isolated by heat-resistant polyalfin film, used in industrial kitchens to shrink-wrap food. In contrast to polyester shrink-wrap, polyalfin film does not melt or release gas in the vacuum chamber. The shrink-wrapping of stub-mounted specimens in polyalfin film reduced the likelihood of costly specimen chamber contamination. Thereafter, the wrapping was carefully heat-sealed to reduce specimen bulk and promote ease of further examination. When the specimen was ready for microscopy, a window was opened over the area of interest by careful cutting of the wrapping, leaving a hole for specimen access. A thorough dusting of particles attached to the wrapping is necessary immediately preceding insertion of the specimen into the specimen chamber, particularly if the fossil has remained in storage for some time.

### Measurement and Anatomical Conventions

**Figure 2:**
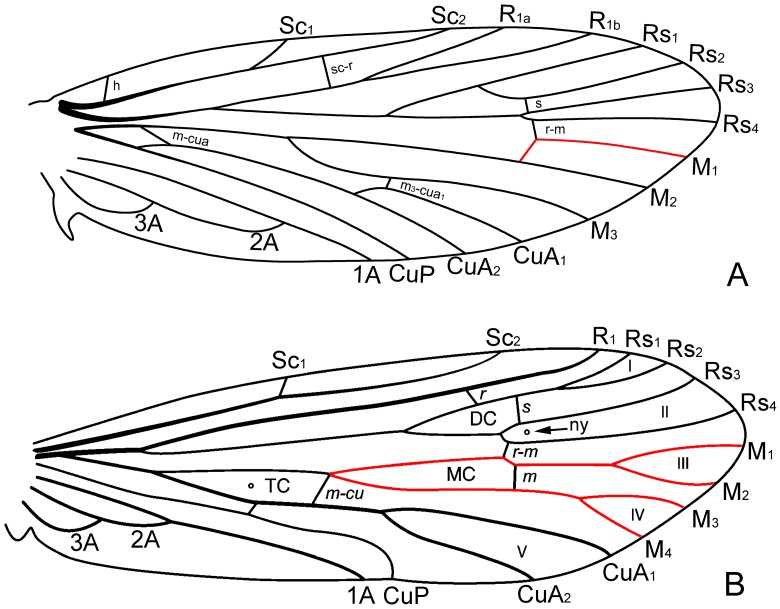
A hypothetical forewing of the Lepidoptera and Trichoptera. (A), A hypothetical forewing of the Lepidoptera modified after Kristensen & Skalski [15]; red line indicates that M_1_, after separation from M_2,_ subtends an angle greater than 60 degrees and is sharply angulate at the junction with the r–m crossvein. (B), A hypothetical forewing of the Trichoptera modified after Holzenthal *et al*. [36]; red line indicates the separation of M_1+2_, and M_1_ consisting of smooth and sublinear veins. Abbreviation: ny, nygmata.

Measurements, including body length and width, wing length and width, and wing index, follow Kristensen and Nielsen [34]. The wing index is defined as the ratio of wing width/wing length. All measurements are given in millimeters (mm). Amphiesmenopteran and lepidopteran apomorphies are taken from Kristensen and Skalski [15] and Huang *et al*. [25]. Family-level classification follows Nieukerken *et al*. [1]. Wing venation nomenclature is based on Wootton [35]. A proposed hypothetical forewing for Lepidoptera, modified from Kristensen and Skalski [15], is illustrated in Fig. 2A. In contrast, a hypothetical forewing for Trichoptera, modified from Holzenthal *et al*. [36], is presented in Fig. 2B. Wing veins are given as combinations of vein abbreviation and the number of vein branches. For example, Rs–4 means that the Rs has four branches; Sc–1/2 indicates that the Sc has one or two branches. Wing vein forking of branches are described as occurring at the same level (equidistant) or at a different level (variable distance) from the base of the wing. The Lepidoptera ovipositor is defined as a composite of abdominal segments VIII, IX+X and associated intersegmental membranes. Additional abbreviations of morphology are provided in the figure captions.

### Etymological Sources

The primary source for Latin and Greek etymology is Simpson [37], buttressed by Brown’s compendium [38]. Linnaean designations of genus and species names were configured to be consistently either Latin or Greek, avoiding combinatorial forms with both languages in the same word.

### Repository

All type specimens described in this report are deposited in the Key Laboratory of Insect Evolution and Environmental Change, College of Life Sciences, Capital Normal University, Beijing, China.

### Nomenclatural Acts

The electronic edition of this article conforms to the requirements of the amended International Code of Zoological Nomenclature, and hence the new names contained herein are available under that Code from the electronic edition of this article. This published work and the nomenclatural acts it contains have been registered in ZooBank, the online registration system for the ICZN. The ZooBank LSIDs (Life Science Identifiers) can be resolved and the associated information viewed through any standard web browser by appending the LSID to the prefix “http://zoobank.org/”. The LSID for this publication is: urn:lsid:zoobank.org:pub: AC86AB61-87FC-4353-96BB-AA03231E3BA5. The electronic edition of this work was published in a journal with an ISSN, and has been archived and is available from the following digital repositories: PubMed Central, LOCKSS (www.lockss.org).

## Results

### Taxonomy

Class Insecta Linnaeus, 1758.

Order Lepidoptera Linnaeus, 1758.

Key to the extant and fossil families of lepidoptera

**1.** Maxillary galeae forming a proboscis ……………….Glossata

**–**Maxillary galeae not forming a proboscis……………………**2**

**2.** M 3-branched………………………………………………**3**

**–** M 4-branched…………………………………………………**5**

**3.** Anterior apophyses present…………….Eolepidopterigidae

**–** Anterior apophyses absent…………………………………**4**

**4.** Sc forked; crossvein present between Sc and R; pterostigma   absent….……………………………………Micropterigidae

**–** Sc unforked; crossvein absent between Sc and R; pterostigma   present…………………………………….Heterobathmiidae

**5.** Metatibia without medial spur; R_1_ forked.…………………  …………….………………Ascololepidopterigidae fam. nov.

**–**Metatibia with medial spurs; R_1_ unforked, …………..………**6**

**6.** Mesotibia with medial spurs; crossvein m-cua

 and crossvein cua–cup present…...………….Agathiphagidae

**–** Mesotibia without medial spur; crossvein m-cua

 and crosvein cua–cup absent……………Mesokristenseniidae

Suborder Eolepidopterigina Rasnitsyn, 1983.

Family Eolepidopterigidae Rasnitsyn, 1983.

#### Type genus

*Eolepidopterix* Rasnitsyn, 1983 [4].

#### Emended diagnosis

Head with mandibulate mouthparts. Antennae filiform, less than half the length of forewing. Pronotum longer than one third the mesonotum length. Foretibia with an epiphysis. Mesotibia with one pair of spurs; metatibia with two pairs of spurs. Both pairs of wings homoneurous (the venation of the forewings and hind wings alike). Wings covered with scales. Forewing with jugum and number of vein branches as follows: Sc-1/2, R_1_-1/2, Rs-4, M-3, CuA-2, CuP-1, and A-2/3. Ovipositor with long apophyses anteriores; papillae anales long, non-piercing.

The synapomorphies for the Eolepidopterigidae are the mesotibia with a pair apical spurs, metatibia with a pair medial spurs and a pair apical spurs, wings homoneurous, forewing M vein 3-branched, and ovipositor with long apophyses anteriores.

This family is defined as a clade in Amphiesmenoptera, which have three branches of the M vein in the hind wing and the anterior margin of female abdominal segments VIII and IX with long, rod-like apodemes. This family can be assigned to Lepidoptera by the following characters: foretibia with an epiphysis, mesotibia lacking medial spurs, wings covered with scales, and forewing M vein 3-branched.

#### Distribution

China, Russia, Kazakhstan, Brazil.

#### Genera included

*Akainalepidopteron* gen. nov.; *Daiopterix* Skalski, 1984 [23]; *Dynamilepidopteron* gen. nov.; *Eolepidopterix* Rasnitsyn, 1983 [4]; *Gracileopterix* Martins-Neto & Vulcano, 1989 [39]; *Grammikolepidopteron* gen. nov.; *Longcapitalis* gen. nov.; *Netoxena* Sohn, 2012 [3] ( =  *Xena* Martins-Neto, 1999 [24]); *Palaeolepidopterix* Kozlov, 1989 [40]; *Petilicorpus* gen. nov.; *Quadruplecivena* gen. nov.; *Seresilepidopteron* gen. nov.; *Undopterix* Skalski, 1979 [10].

#### Key to the known genera of eolepidopterigidae

**1**. Body longer than 9 mm; length of forewing nearly 10 mm;

 hind wing with crossvein sc–r_1_ near the R_1_ furcation  …………………...………….………*Quadruplecivena* gen. nov.

**–** Body less than 8 mm; length of forewing less than 9 mm;

 hind wing lacking crossvein sc–r_1_…………..……………….**2**

**2**. Forewing with Sc not forked…………………..……………**3**

**–** Forewing with Sc forked…………………………………….**6**

**3**. Forewing with R_1_ not forked…………………………….…**4**

**–** Forewing with R_1_ forked…………………………………….**5**

**4**. Anal veins of a single-Y configuration (Fig. 1E)….... *Netoxena*

**–** Anal veins of a double-Y configuration………………………  ……………………………...….*Grammikolepidopteron* gen. nov.

**5**. Forewing less than 3 mm; Rs_1+2_ stalked,

 ca. 1/3 of its total length……………………...…*Gracileopterix*

**–** Forewing longer than 5 mm, Rs_1+2_ stalked,

 more than half of its total length………….....*Palaeolepidopterix*

**6**. Forewing with R_1_ not forked.………….*Longcapitalis* gen. nov.

**–** Forewing with R_1_ forked………………………………....….**7**

**7**. Sc branching near the base of forewing…..…….*Eolepidopterix*

**–** Sc branching distally…………………………………....……**8**

**8**. Rs_3+4_ forking beyond the Rs_1+2_ furcation………………….**9**

**–** Rs_3+4_ and Rs_1+2_ forking at the same level……………...….**11**

**9**. Forewing with Rs_3+4_ forking level with M_1+2_ furcation  …………………..…….……….…….…. *Petilicorpus* gen. nov.

**–** Forewing with Rs_3+4_ forking beyond M_1+2_ fork (Fig. 1D)  ……………………………………………….…………….**10**

**10**. Humeral vein present on forewing…...………….*Undopterix*

**–** Humeral vein absent on forewing………………..…*Daiopterix*

**11**. Hind wing with spines on several veins.……………………  ……………..…………….….…….*Akainalepidopteron* gen. nov.

**–** Hind wing lacking spines………………………………..…**12**

**12**. Anterior margins of fore and hind wings lacking cilia;

 metatibia with irregularly arranged spines…………………  ……………...……………………*Dynamilepidopteron* gen. nov.

**–** Anterior margins of fore and hind wings with cilia;

 metatibia lacking spines…………….*Seresilepidopteron* gen. nov.

#### Remarks

Kristensen [41] considered Skalski’s family assignment of all species, except *Eolepidopterix jurassica*, to the Eolepidopterigidae [7] unconvincing due to lack of definitive apomorphies. Subsequently, Nieukerken *et al*. [1] placed only *E. jurassica* in the Eolepidopterigidae. We assign all the above-mentioned genera to this family for the following reasons. Skalski erected the genus *Undopterix* in Micropterigidae [10], with the type species as *U. sukatsheva* Skalski 1979. Another supposed congener was added, *U. caririensis* Martins-Neto & Vulcano, 1989 from the Crato Formation, Brazil [10], [39]. The taxonomic position of this genus is controversial: some authors have placed it in Micropterigidae [10]; some assign it to the Undopterigidae [16]; whereas others considered it as Eolepidopterigidae [4], [7]. Yet, *Undopterix sukatsheva* Skalski, 1979, exhibits no autapomorphies of the Micropterigidae [34]. The long apophyses of the last several abdominal segments are comparable to those present in the Eolepidopterigidae [4]. For wing venation, *Undopterix* shows similarity to Eolepidopterigidae, especially in the hind wing. *Undopterix* has R_1_ forked, which is rare in Micropterigidae, but present in some members of Eolepidopterigidae, such as *Daiopterix* Skalski [23], *Seresilepidopteron* gen. nov., *Akainalepidopteron* gen. nov., *Quadruplecivena* gen. nov., and *Petilicorpus* gen. nov. Consequently, we believe it is more appropriate to place *Undopterix* in the Eolepidopterigidae. We agree with Skalski [7], followed by Sohn *et al*. [3], in considering the Undopterigidae a synonym of the Eolepidopterigidae. In addition, the Eolepidopterigidae are believed to have affinities with the Agathiphagidae [7], based on an ovipositor bearing long apophyses that is not present in Micropterigidae and Heterobathmiidae [34]. When *Gracileopterix* was erected, it was not assigned to a family [39], and because of its great similarity to *Undopterix*, we tentatively place this genus in Eolepidopterigidae.

### *Seresilepidopteron* Zhang, Shih, Labandeira & Ren gen. nov

urn:lsid:zoobank.org:act:5AB2FE11-A3A1-4593-B01B-29E7C8E25C49.

#### Type species

*Seresilepidopteron dualis* Zhang, Shih, Labandeira & Ren sp. nov.

#### Etymology

The generic name, *Seresilepidopteron*, is derived from the Greek, *Seres* (Σηρες), the ancient Greek (and later similar Latin) name for northwestern China and its inhabitants, also referring to the land where silk originates; and *lepidos*, the Greek word for “scale” or “flake”; and *pteron*, Greek, for “wing” or “fin.” The root, *lepido*, also refers to the Lepidoptera, the ordinal affiliation of this genus. The gender is masculine.

#### Diagnosis

Mesothorax slightly longer and wider than metathorax. All legs lacking spines. Forewing Sc and R_1_ forked, all furcations of Rs_1+2_, Rs_3+4_ and M_1+2_ at the same level with each other. Humeral vein, sc–r crossvein and crossvein between R_1_ and Rs present; three anal veins looping into a double-Y configuration; jugum present but short, lacking long setae. Hind wing Sc not forked; R_1_ forked; sc–r crossvein present.

An Eolepidopterigidae affiliation is supported by: 1), forewing with jugum; 2), M 3-branched; and 3), ovipositor segments with long apophyses anteriores.

#### Distribution

Inner Mongolia Autonomous Region, China.

#### Comparison

The venation of *Seresilepidopteron* resembles that of *Daiopterix*, but *Seresilepidopteron* differs from the latter by three anal veins looping into a double-Y configuration (vs. only two anal veins looping into a single-Y configuration). *Seresilepidopteron* resembles *Undopterix*, but it differs from the latter by: 1), having sc–r and r crossveins (vs. lacking sc–r and r crossveins); 2), the presence of three anal veins; and 3), a double**-**Y configuration in the forewing (vs. two anal veins).

### *Seresilepidopteron dualis* Zhang, Shih, Labandeira & Ren sp. nov. (Figs. 3, 4)

urn:lsid:zoobank.org:act:C4F29D5D-B13E-4BF3-84A4-A11E41319C4F.

#### Etymology

The specific name is derived from the Latin, *dualis* (dual, twice).

#### Type materials

Holotype: CNU-LEP-NN-2006-001P/C (part and counterpart); ♂; well preserved head, thorax, abdomen, fore and hind wings, parts of legs. Paratype: ♀; CNU-LEP-NN-2006-002; excellently preserved body and forewings.

#### Locality and horizon

These specimens were collected from Daohugou Village, Shantou Township, Ningcheng County, Inner Mongolia Autonomous Region, China. The age is latest Middle Jurassic, near the Callovian–Oxfordian boundary.

#### Diagnosis

Same as generic diagnosis.

#### Description

**Figure 3:**
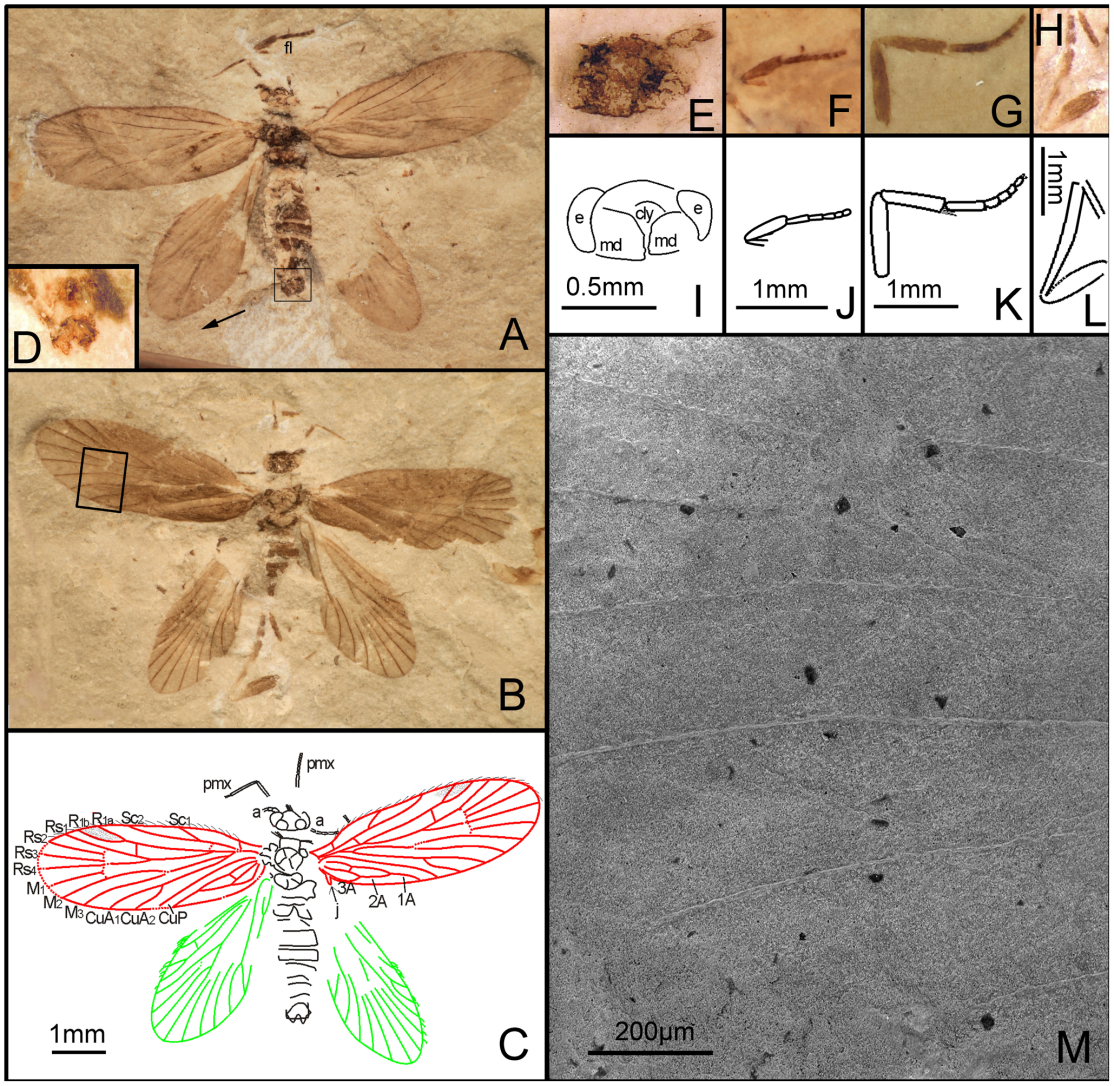
*Seresilepidopteron dualis* gen. et sp. nov. Male, holotype, CNU-LEP-NN-2006-001P/C. (A), Fossil specimen (counterpart). (B), Fossil specimen (part). (C), Camera lucida drawing of (A), showing overall habitus. (D), Genitalia (counterpart), outlined at (A). (E), Head and associated features (part). (F), Foreleg (counterpart). (G), Middle leg (counterpart). (H), Hind leg (part). (I) to (L), Camera lucida drawings of head (I), foreleg (J), middle leg (K), and hind leg (L). (M), SEM micrograph of left forewing venation, enlarged from the rectangular template in (B). Abbreviations: a, antennae; cly, clypeus; e, eye; fl, foreleg; j, jugum; md, mandible; pmx, maxillary palpus.

Male (Fig. 3). Head partially preserved; antennae filiform; compound eyes widely separated. Portions of two mandibles and clypeus visible on ventral view of head (Figs. 3E, I). Last segment of a maxillary palpus visible, with sparse setae. Mesothorax slightly longer and wider than metathorax. No traces of setal warts on thorax. Almost all segments of abdomen preserved; genitalia visible (Fig. 3D).

Foreleg (Figs. 3F, J) detached and separated ca. 0.8 mm from the head of the insect, with faint trace of epiphysis; tarsi 5-segmented. Midleg (Figs. 3G, K) well preserved, covered with dense hairs, but detached ca. 5.0 mm from the insect. Tarsi 5-segmented, with spines; apical segment with a pair of tarsal claws. A pair of apical spurs apparent on the mesotibia (Fig. 3K). Femur of hindleg robust, detached (Figs. 3H, L); ca. 3.1 mm apart from the posterior margin of metathorax.

Forewing (Fig. 3C) with a humeral vein; costal margin bearing sparse cilia. Sc forked, stalked, gently curved; Sc_2_ reaching proximal margin of pterostigma. R_1_ forked, both branches of R_1_ extend to pterostigma. Rs 4-branched; Rs_4_ to the apex of forewing; Rs_1+2_, Rs_3+4_, and M_1+2_ furcations arise at same level in the wing. Crossvein sc–r oblique, proximal to the R_1_–Rs furcation. Crossvein r located midway between R_1_ furcation and Rs furcation. M 3-branched, the hyaline zones surround the r–m crossvein at Rs_1+2_, Rs_3+4_ and M_1+2_ furcations. CuA bifurcated; CuP simple, slightly curved terminally. Stem of M divergent from stem of R basally, just beyond humeral vein. CuA divergent from M slightly beyond R–M furcation; R forks just after M–CuA furcation. Three anal veins looping into a double-Y configuration. Crossvein between 1A and 2A present. A short, digitate jugum present in the basal posterior margin of forewing, lacking long setae. Hind wing venation (Fig. 3C) resembles forewing except Sc not forked; sc–r crossvein present proximally to R_1_ furcation. Costal margin bears long bristles. Anal area poorly preserved.

**Figure 4:**
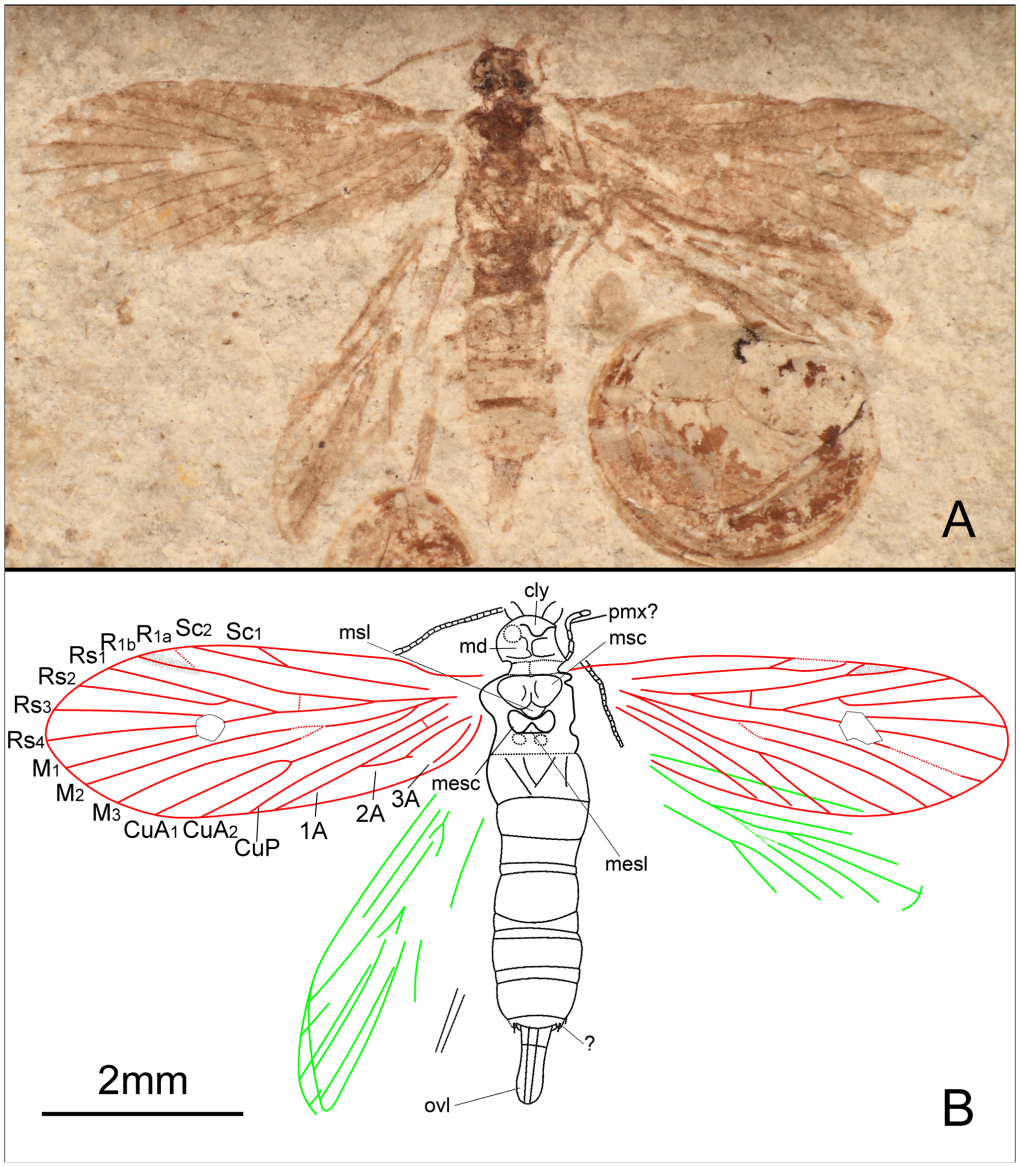
*Seresilepidopteron dualis* gen. et sp. nov. Female, paratype, CNU-LEP-NN-2006-002. (A), Fossil specimen, with a conchostracan at lower-right. (B), Camera lucida drawing of (A), showing overall habitus. Abbreviations: cly, clypeus; md, mandible; msc, mesoscutum; msl, mesoscutellum; mesc, metascutum; mesl, metascutellum; ovl, ovipositor lobes; pmx, maxillary palpus.

Paratype: Female (Fig. 4). Head with large mandibles; antennae filiform, shorter than forewing; robust scape. Maxillary palpus 5-segmented; fourth segment longest. Mesothorax longer and wider than metathorax. Mesoscutum, mesoscutullum, metascutum and metascutullum well preserved. All abdominal segments visible; ovipositor long, well developed, with a pair of inner apophyses.

Wing venation (Fig. 4B) of female similar to male, except for the following differences: 1), Sc_2_ extends to anterior wing margin at midlength in male, after midlength in female; 2), R_1_ furcation in female closer to the apex than that in male; 3), in female, crossvein between R_1_ and Rs located before Rs furcation on right wing, but in male after Rs furcation; 4), in female, cup–a crossvein present but no crossvein in anal area; and 5), in male, cup–a crossvein absent, but crossvein in anal area present in the female.

#### Measurements (in mm)

CNU-LEP-NN-2006-001P/C: body length 4.3; width 0.9. Forewing length 4.7; width 1.7. Hind wing length 3.8; width 1.7. For CNU-LEP-NN-2006-002: body length 5.0 and width 1.1; forewing length 4.5 and width 1.8; and hind wing length 3.9.

#### Remarks

Because there are many similarities in provenance and morphology between these two specimens from the same locality, we consider these two specimens to be conspecific male and female. The minor differences in the wing venation between male and female could be attributable to either individual variation or sexual dimorphism.

### *Akainalepidopteron* Zhang, Shih, Labandeira & Ren gen. nov

urn:lsid:zoobank.org:act:F017D748-9F58-407E-87AF-75063A531CCA.

#### Type species

*Akainalepidopteron elachipteron* Zhang, Shih, Labandeira & Ren sp. nov.

#### Etymology

The generic name *Akainalepidopteron* is derived from the Greek *akaina*, (spine, thorn), referring to the piliform scales on the hind wing veins of this species; and “*lepidos*,” the Greek name for “scale” or “flake”; and “*pteron*,” Greek for “wing” or “fin.” The root, *lepidos*, also refers to the Lepidoptera, the ordinal affiliation of this genus. The gender is masculine.

#### Distribution

Inner Mongolia Autonomous Region, China.

#### Diagnosis

Mesotibia with few short spines; metatibia with robust spines. Forewing and hind wing bear cilia on the anterior margin. Humeral vein present in forewing. Sc and R_1_ forked; all furcations of Rs_1+2_, Rs_3+4_, and M_1+2_ ca. at same level with each other. Hind wing Sc not forked. R_1_ forked; furcations of Rs_1+2_, Rs_3+4_, and M_1+2_ almost at the same level. Long, piliform scales present on several veins of hind wing. Crossvein cua–cup present.

An Eolepidopterigidae affiliation is supported by: 1), antennae less than half the length of forewing; 2), wings homoneurous; 3), M 3-branched; and 4), crossvein absent between Sc and R.

#### Comparison

The diagnostic autapomorphy of *Akainalepidopteron* consists of piliform scales present on the veins of the hind wing, a character not known to exist for any other lepidopteran fossil species. *Akainalepidopteron* exhibits a great similarity to *Seresilepidopteron* in venation, but differs in the position of some crossveins. The differences are: 1), in the forewing, *Akainalepidopteron* lacks sc–r and r crossveins (vs. retention of these crossveins); 2), in the hind wing, the cua–cup crossvein is present in *Akainalepidopteron* (vs. absence); and 3), the sc–r crossvein is absent in *Akainalepidopteron* (vs. presence).

*Akainalepidopteron* also is similar to *Daiopterix*, especially to *D. olgae* that has wing cilia and metatibial spines. *Akainolepidopteron* is distinguished from *Daiopterix* by the following characters: 1), the cup–a_1_ crossvein is absent in *Akainalepidopteron* (vs. present); and 2), the humeral vein is present in *Akainalepidopteron* (vs. absent).

Compared with *Undopterix*, *Akainalepidopteron* is different in that: 1), it has forking of Rs_1+2_ at the same level as forking of Rs_3+4_ (vs. a stem Rs_1+2_ only half length of the stem Rs_3+4_); 2), the anterior margin of forewing and hind wing bears cilia in *Akainalepidopteron* (vs. lacking cilia); and 3), the metatibiae has spines in *Akainalepidopteron* (vs. lacking spines).

### *Akainalepidopteron elachipteron* Zhang, Shih, Labandeira & Ren sp. nov. (Figs. 5, 6)

urn:lsid:zoobank.org:act:6D4EBD55-F1FA-4F7F-BA7E-B95C0D0C0F65.

#### Etymology

The specific name is derived from the Greek, *elachys* (short, small), and *pteron (*wing, fin), referring to the wing of this species that is shorter than its body length.

#### Type materials

Holotype: CNU-LEP-NN-2012-024; ♀; well preserved fore and hind wings; anal area covered by body and portions of the legs. Paratypes: CNU-LEP-NN-2012-023; ♀; well preserved legs; anal area covered by body and part of hind wing; and CNU-LEP-NN-2012-026; sex unknown; a well preserved forewing; anal area poorly preserved.

#### Locality and horizon

These specimens were collected from Daohugou Village, Shantou Township, Ningcheng County, Inner Mongolia Autonomous Region, China. The age is latest Middle Jurassic, near the Callovian–Oxfordian boundary.

#### Diagnosis

Same as generic diagnosis.

#### Description

Head length subequal to width, invested with dense setae on the anterior margin. Antennae filiform, tapered to apex; length of segments equal to their diameter. Eyes oval, with sparse setae on the outer margin.

Forecoxae more robust than forefemora; femora ca. 1.6 times as long as foretibiae, slightly longer than foretarsi. Epiphyses indiscernible. Mesocoxae more slender than forecoxae; mesofemora longer than mesotibia; two long spines present at the end of mesofemora; mesotibia with spines. Metafemora robust and short, less than half the length of metatibia; metatibia with irregularly arranged spines at the distal part, and with one pair medial spurs and one pair apical spurs. All tarsal segments with terminal spinules.

**Figure 5:**
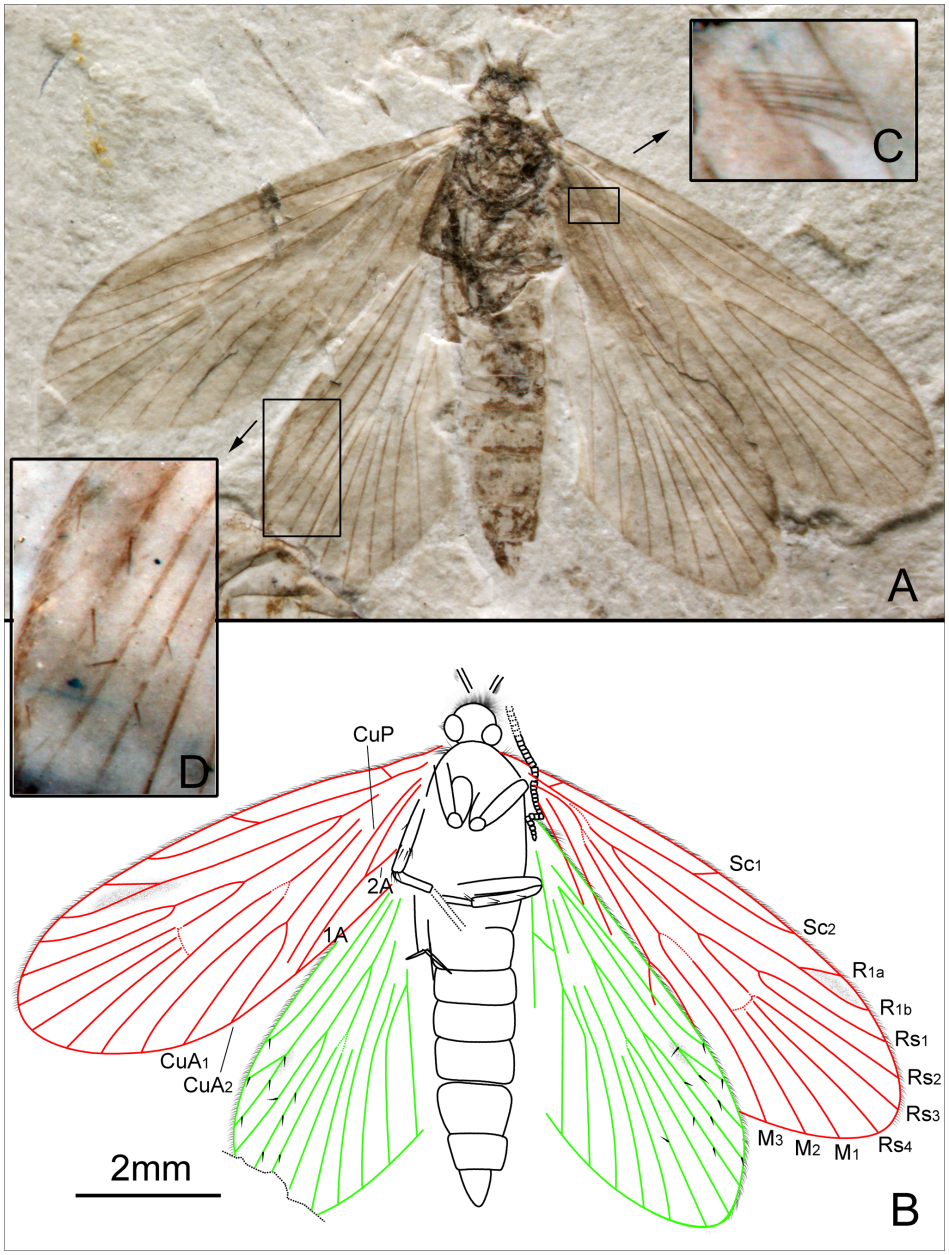
*Akainalepidopteron elachipteron* gen. et sp. nov. Female, holotype, CNU-LEP-NN-2012-024. (A), Fossil specimen. (B), Camera lucida drawing of (A), showing overall habitus. (C), A cluster of frenular bristles (wing coupling apparatus) on hind wing, outlined at the upper-right rectangular template in (A). (D), Piliform scales on hind wing, outlined at the lower-left rectangular template in (A).

Forewing ca. 2.9 times as long as wide; anterior margin bearing cilia; pterostigma present. Humeral vein present. Sc forked from distal 1/3 of stem; Sc_2_ extending to the costal margin at 2/3 length of the wing from its base. R_1_ forked distally; Rs 4-branched; Rs_4_ ending slightly below the apex of forewing; Rs_1+2_ stalked ca. 0.3–0.5 of their total length; stem Rs_1+2_ subequal to stem Rs_3+4_. M 3-branched. Hyaline zones surrounding r–m crossvein, at Rs_3+4_ and M_1+2_ furcations. CuA bifurcated; CuP simple; anal area not well preserved. CuA divergent from M. R forks immediately after M–CuA furcation. Hind wing ca. 2.4 times as long as wide; anterior margin bearing cilia, and a cluster of ca. 7 frenular bristles arising near the base of C on hind wing (Fig. 5C). Sc not forked, extending 2/3 length of the wing from its base. R_1_ forked; Rs 4-branched; Rs_1+2_ forked at the same level with Rs_3+4_. M 3-branched. CuA bifurcated; CuP simple. Crossvein cua–cup slanted obliquely. Piliform scales ranging from 0.12 to 0.21 mm in length, present on veins Sc, R_1a_, R_1b_, Rs_1_, Rs_2_, Rs_3_ and Rs_4_ (Figs. 5 B,D).

**Figure 6:**
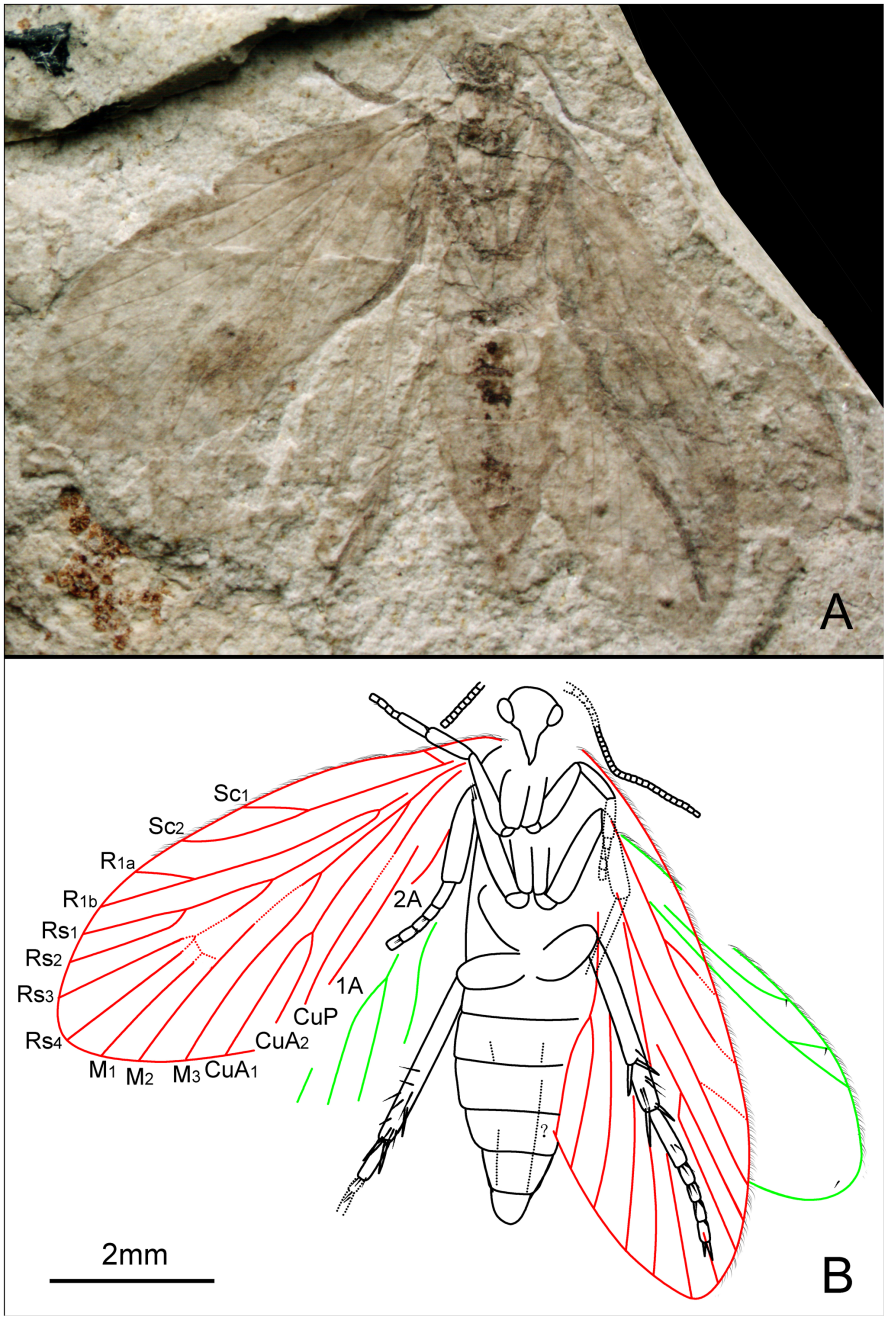
*Akainalepidopteron elachipteron* gen. et sp. nov. Female, paratype, CNU-LEP-NN-2012-023. (A), Fossil specimen. (B), Camera lucida drawing of (A), showing overall habitus.

Female genitalia with short ovipositor; apophyses probably present.

#### Measurements (in mm)

CNU-LEP-NN-2012-024: body length 7.2 and width 1.5; forewing length 7.3 and width ca. 2.5; hind wing length 6.2 and width ca. 2.6. CNU-LEP-NN-2012-023: body length 6.3 and width 1.3; forewing length ca. 6.0 and width ca. 2.4; Hind wing length ca. 5.3. CNU-LEP-NN-2012-026: forewing length ca. 11.1, width 5.1.

#### Remarks

There are three forms of wing-coupling in Lepidoptera: the jugate, frenate and amplexi conditions. The frenular bristles on the anterior margin of the hind wing of CNU-LEP-NN-2012-024 (Fig. 5C) probably played an important role in wing-coupling. Because the jugum is not discernible in these specimens and the female possesses multiple frenular bristles, we believe that this species probably possessed a modified jugate–frenate form of wing-coupling.

Piliform scales, with lengths between 0.07 and 0.08 mm, are present on some lepidopterans, e.g. *Heterobathmia pseuderiocrania* Kristensen & Nielsen, 1979, with body lengths from 3.2 to 3.6 mm and forewing lengths from 4.5 to 5.0 mm [34]. The scales present on the hind wing veins of CNU-LEP-NN-2012-024 are of similar shape and length (adjusted for lengths of body and wings) to those piliform scales along the veins of *H. pseuderiocrania*.

### *Dynamilepidopteron* Zhang, Shih, Labandeira & Ren gen. nov

urn:lsid:zoobank.org:act:31AD01ED-D78A-45D0-8E80-AB92CC94977F.

#### Type species

*Dynamilepidopteron aspinosus* Zhang, Shih, Labandeira & Ren sp. nov.

#### Etymology

The generic name is derived from the Greek, *dynamis* (power, strength), referring to the robust hind leg of this genus; and *lepidos*, Greek for “scale” or “flake,” also referring to the ordinal name, Lepidoptera, to which this genus belongs; and *pteron*, meaning “wing” or “fin” in Greek. The gender is masculine.

#### Distribution

Inner Mongolia Autonomous Region, China.

#### Diagnosis

Body relatively large, ca. 7 mm long. Metatibia robust, with strong spines. Forewing and hind wing lack cilia on the anterior margin. Humeral vein present in forewing. Sc and R_1_ forked; all furcations of Rs_1+2_ and Rs_3+4_ veins at same level. Hind wing lacking spines.

An Eolepidopterigidae affiliation is supported by: 1), antennae less than half the length of forewing; 2), wings homoneurous; 3), M 3-branched; and 4), crossvein absent between Sc and R.

#### Comparison

*Dynamilepidopteron* is similar to *Akainalepidopteron* in venation, but: 1), the former lacks cilia on the anterior wing margins (vs. both forewing and hind wing with cilia); and 2), the hind wing lacks spines on the veins (vs. spines present on the veins).

### *Dynamilepidopteron aspinosus* Zhang, Shih, Labandeira & Ren sp. nov. (Fig. 7)

urn:lsid:zoobank.org:act:EB75EC3D-5811-43EE-9232-F755D5B1EED8.

#### Etymology

The specific name is derived from the Latin, *aspinosus* (the absence of spines), in reference to the hind wing lacking spines.

**Figure 7:**
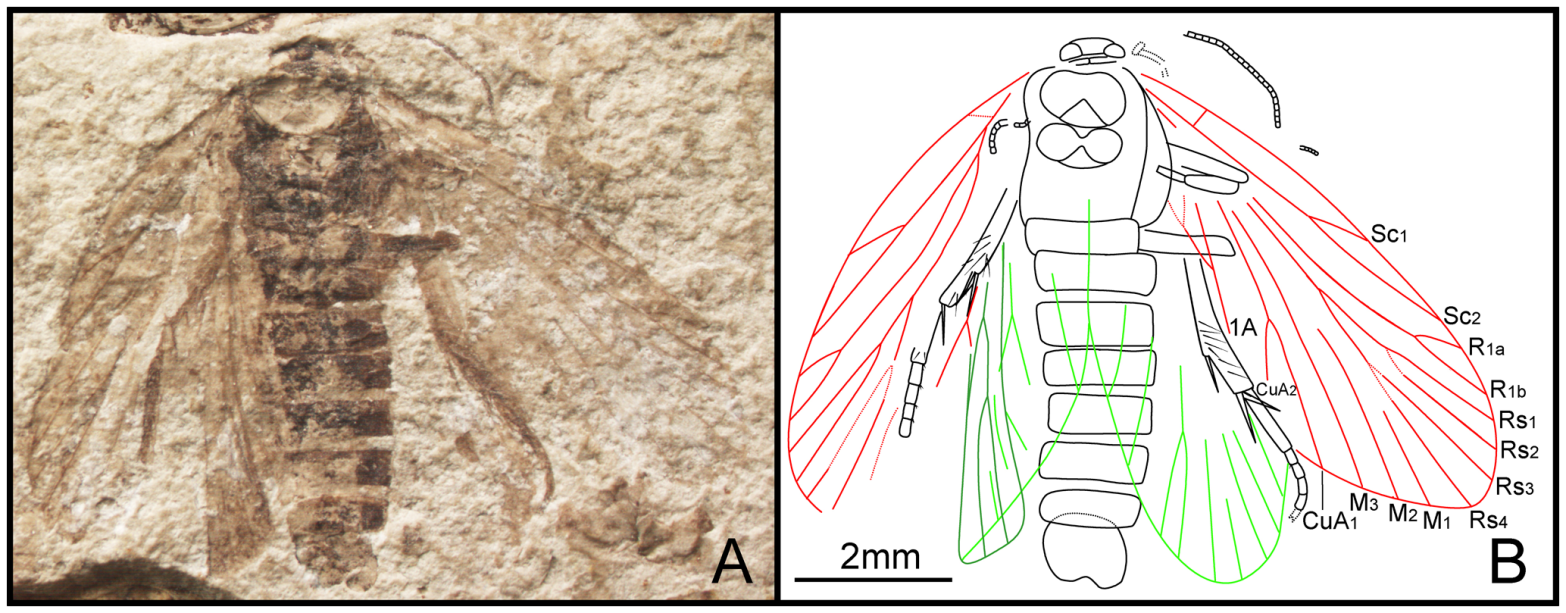
*Dynamilepidopteron aspinosus* gen. et sp. nov. Male, holotype, CNU-LEP-NN-2012-014. (A), Fossil specimen. (B), Camera lucida drawing of (A), showing overall habitus.

#### Type material

Holotype: CNU-LEP-NN-2012-014; ♂; well preserved forewings, thorax and hind legs.

#### Locality and horizon

This specimen was collected from Daohugou Village, Shantou Township, Ningcheng County, Inner Mongolia Autonomous Region, China. The age is latest Middle Jurassic, near the Callovian–Oxfordian boundary.

#### Diagnosis

Same as generic diagnosis.

#### Description

Head short and broad, possibly due to preservation. Antennae filiform; length of segments equal to their diameter basally, the length ca. 1.5 times as long as their diameter distally. Maxillary palpus visible, obtuse aspect apically. Mesoscutum broader than long, posterior edges of mesoscutum concave along midline; mesoscutellum flabellate, relatively large, nearly equal to mesoscutum in length; metascutum dumbbell shaped; metascutellum smaller than mesoscutellum. Metatibia relatively robust; with irregularly arranged spines, bearing two pairs of spurs, one pair apically and the other pair arising slightly beyond middle of tibia; all spurs ca. 1.5–2 times as long as diameter of tibia. Metatarsi 5-segmented; tarsomere I longest, nearly equal to the total length of tarsomeres II, III, IV and V combined; two spinules at the apex of each tarsomere.

Forewing 2.8 times as long as wide. Humeral vein present. Sc forked from 2/5 of the stem length; Sc_1_, extending midway along costal margin; Sc_2_ beyond 2/3 the length of the wing from its base. R_1_ forked distally, slightly beyond Rs_1+2_ furcation; Rs 4-branched; Rs_4_ terminating slightly below the apex of forewing; Rs_1+2_ stalked ca. 0.3–0.4 of their total length; Rs_3_ and Rs_4_ free; stem Rs_1+2_ subequal in length to stem Rs_3+4_. M 3-branched. CuA bifurcated; CuP not preserved. Three anal veins looping into a double-Y configuration.

#### Measurements (in mm)

Body length 7.0; width 1.9. Forewing length 6.7; width ca. 2.4. Hind wing length ca. 5.5.

### *Quadruplecivena* Zhang, Shih, Labandeira & Ren gen. nov

urn:lsid:zoobank.org:act:878C8B3C-643E-4C46-B56A-ABDA90AEB775.

#### Type species

*Quadruplecivena celsa* Zhang, Shih, Labandeira & Ren sp. nov.

#### Etymology

The generic name, *Quadruplecivena*, is derived from the Latin, *quadruplex* (quadruple, fourfold); and *vena* (vein, blood vessel); which jointly refers to the costal margin of hind wing that consists of a Sc, sc–r_1_ crossvein and a R_1a_, forming a quadrate configuration. The gender is feminine.

#### Distribution

Inner Mongolia Autonomous Region, China.

#### Diagnosis

Labial palpus 3-segmented. All legs lacking spines. Anterior margins of fore and hind wings with cilia. Forewing with humeral vein; Sc forked; R_1_ not forked; Rs_1+2_ forking at same level with Rs_3+4_, and before M_1+2_ forking. Hind wing Sc not forked; R_1_ forked; sc–r_1_ crossvein present.

An Eolepidopterigidae affiliation is supported by: 1), antennae less than half the length of forewing; 2), mesotibia with a pair of spurs; 3), wings homoneurous; and 4), M 3-branched.

#### Comparison

*Quadruplecitvena* differs from *Seresilepidopteron* in the following characters: in the forewing, 1), the M_1+2_ distinctly extends beyond the Rs_3+4_ furcation (vs. the M_1+2_ and Rs_3+4_ furcations occurring at the same level); 2), the sc–r crossvein is absent (vs. presence of the sc–r crossvein); and 3), hind wing sc–r_1_ crossvein is near the R_1_ furcation (vs. the sc–r crossvein considerably removed from the R_1_ furcation). *Quadruplecivena* differs from *Akainalepidopteron* in the following characters: 1), the M_1+2_ furcation distinctly extends beyond the Rs_3+4_ furcation (vs. the M_1+2_ and Rs_3+4_ furcations occurring at the same level); 2), the M_1_ is not sharply angulate at the junction with the r–m crossvein (vs. M_1_ sharply angulate at the junction with r–m crossvein); 3), the hind wing lacks spines (vs. hind wing with spines); 4), the sc–r_1_ crossvein is present and the cua–cup crossvein absent (vs. the sc–r_1_ crossvein absent, and the cua–cup crossvein present); and 5), the legs lack spines (vs. legs with irregularly arranged spines).

### *Quadruplecivena celsa* Zhang, Shih, Labandeira & Ren sp. nov. (Figs. 8, 9)

urn:lsid:zoobank.org:act:1EBA2307-D14A-4B56-93B2-2069AA3BA10D.

#### Etymology

The specific name is derived from the Latin *celsa* (eminent, noble) in reference to the labial palpi, which are prominent and resemble a crown, providing a noble or regal appearance.

**Figure 8:**
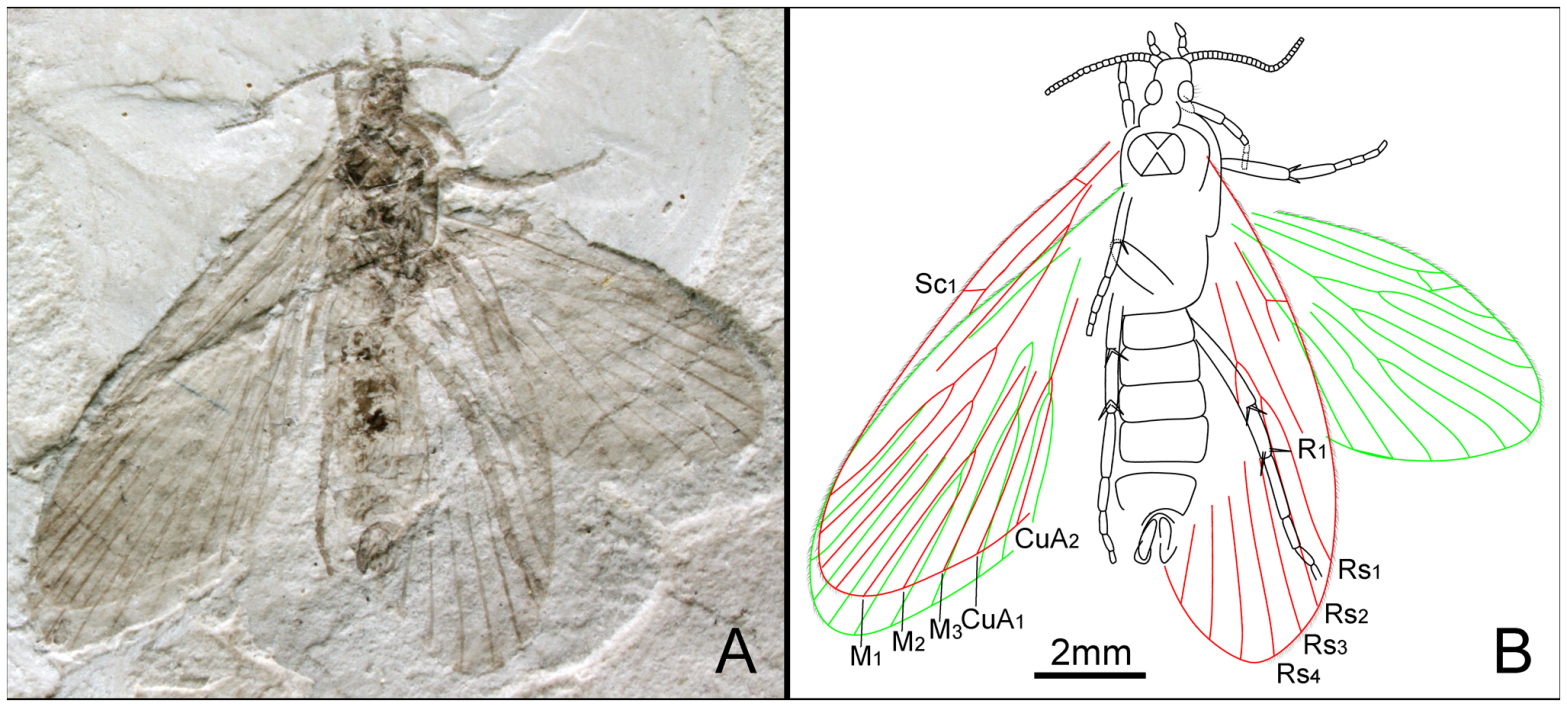
*Quadruplecivena celsa* gen. et sp. nov. Male, holotype, CNU-LEP-NN-2012-027. (A), Fossil specimen. (B), Camera lucida drawing of (A), showing overall habitus.

#### Type material

Holotype: CNU-LEP-NN-2012-027; ♂; consisting of a well-preserved head, with antennae and labial palpi; well-preserved legs. Left fore- and hind wings overlapping. The right fore- and hind wings are partially preserved; the anal area is poorly preserved. The left hind wing is unusually longer than the right hind wing, probably due to deformation of the matrix or actual wing asymmetry of this particular individual.

#### Locality and horizon

This specimen was collected from Daohugou Village, Shantou Township, Ningcheng County, Inner Mongolia Autonomous Region, China. The age is latest Middle Jurassic, near the Callovian–Oxfordian boundary.

#### Diagnosis

Same as generic diagnosis.

#### Description

**Figure 9:**
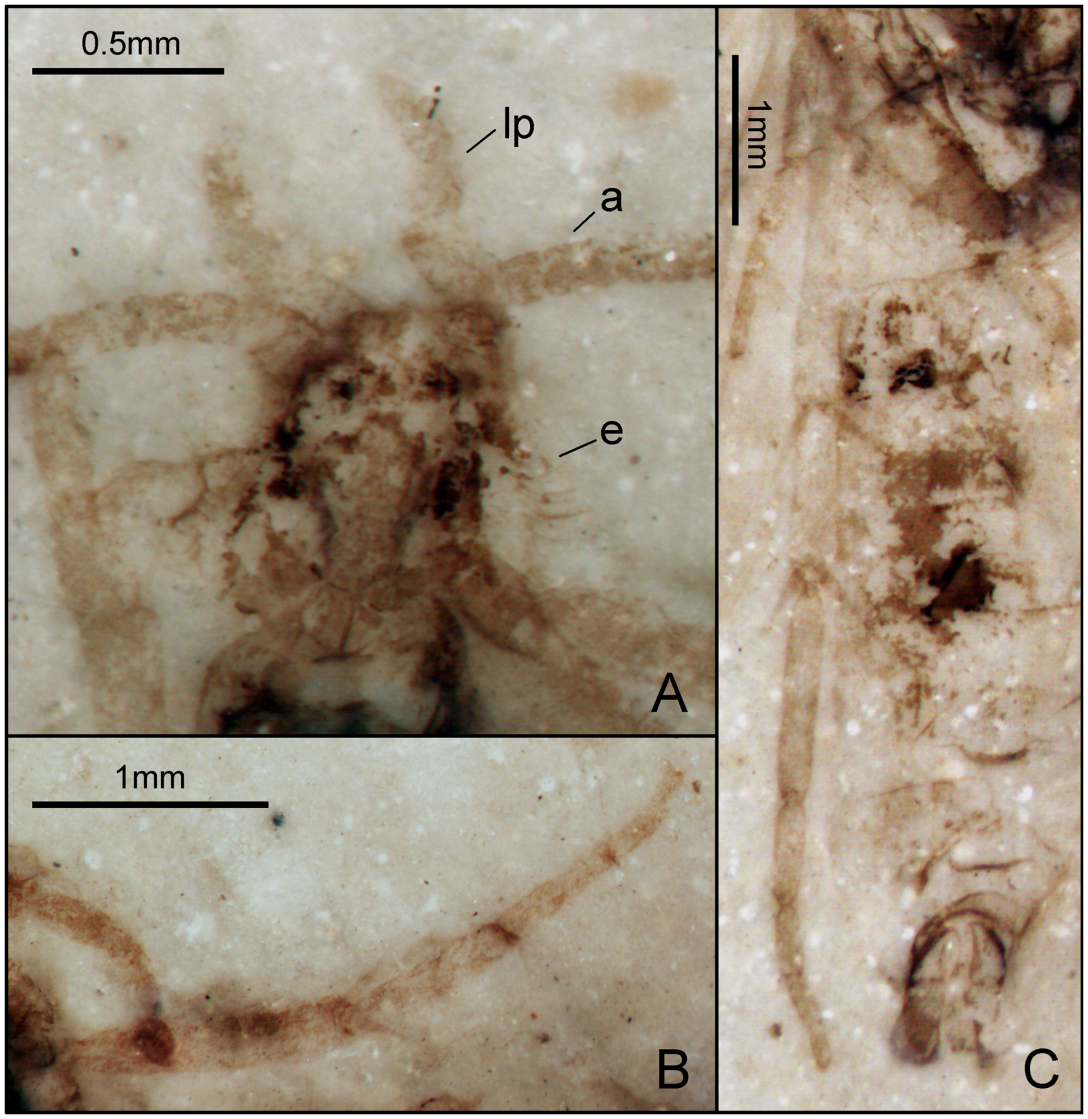
*Quadruplecivena celsa* gen. et sp. nov. CNU-LEP-NN-2012-027. (A), Head of fossil specimen. (B), Right mid leg. (C), Hind leg and abdomen. Abbreviations: a, antennae; e, eye; lp, labial palpus.

Male. Antennae filiform, tapered to apex, ca. 1/3 as long as forewing; length of segments shorter than their diameter basally, subequal to their diameter distally. Compound eyes bearing long sparse setae. Labial palpi 3-segmented (Fig. 9A), the last two segments nearly equal in length; apical palpal segments tapered toward apex. Mesothorax with large prescutum; anterior and posterior edge of mesonotum distinctly concave along midline; mesoscutellum triangular. Foretibial epiphysis indistinct. Mesotibia with one pair of apical spurs (Fig. 9B). Metatibia with one pair of medial spurs and one pair of apical spurs (Fig. 9C). Metatarsi 5-segmented, tarsomere I ca. 1.9 times as long as tarsomere II; tarsomere II ca. 1.6 times as long as tarsomere III; tarsomere III subequal to tarsomere IV, and slightly longer than tarsomere V. All tarsal segments lacking terminal spinules.

Forewing 3 times as long as wide; anterior margin bearing cilia. Humeral vein present. Sc forked. Rs 4-branched; Rs_1+2_ stalked ca. 0.2 of their total length; Rs_1+2_ forked at the same level as Rs_3+4_ furcation; Rs_4_ and M_1_ respectively above and below wing apex. M 3-branched; M_1_ and M_2_ stalked; M_1+2_ forked beyond Rs_3+4_ furcation. CuA bifurcated. Anal veins not preserved. In hind wing, Sc not forked. R_1_ forked; crossvein sc–r_1_ present and slightly curved and oblique, originating near end of Sc and terminating near R_1_ furcation; Rs 4-branched; stem Rs_1+2_ slightly longer than Rs_3+4_. M 3-branched; M_1+2_ forked beyond Rs_3+4_ furcation; M_3_ curved at 2/3 the length of vein M_3_ from its base. CuA bifurcated.

#### Measurements (in mm)

Body length ca. 9.5; width 1.9. Forewing length 9.8; width ca. 3.3. Hind wing length ca. 7.8 (right wing) and 9.8 (left wing); width 3.8 (right wing) and 3.4 (left wing).

### *Petilicorpus* Zhang, Shih, Labandeira & Ren gen. nov

urn:lsid:zoobank.org:act:070D5F91-A174-4BE8-A0B1-503475BF1817.

#### Type species

*Petilicorpus cristatus* Zhang, Shih, Labandeira & Ren sp. nov.

#### Etymology

The generic name is derived from the Latin *petilis* (thin, slender), and *corpus* (body, substance), which collectively refers to the gracile body shape of this species. The gender is masculine.

#### Distribution

Inner Mongolia Autonomous Region, China.

#### Diagnosis

Body slender. Anterior margins of fore and hind wing with cilia. In forewing, Sc and R_1_ forked; Rs branched into Rs_1+2_, and Rs_3+4_; Rs_3+4_ forked beyond that of Rs_1+2_; Rs_3+4_ furcation nearly at same level as M_1+2_ furcation; crossvein m present. Hind wing with Sc not forked; R_1_ forked. In female, apophyses well developed.

An Eolepidopterigidae affiliation is supported by: 1), wings homoneurous; 2), M 3-branched; 3), ovipositor segments with long apophyses anteriores; and 4) crossvein absent between Sc and R.

#### Comparison

*Petilicorpus* gen. nov. is assigned to the Eolepidopterigina for having a long apophyses in the abdomen and the M vein 3-branched in the forewing. *Petilicorpus* resembles *Eolepidopterix* in having the presence of a slender body and similar forewing length and antennal length. However, *Petilicorpus* differs from *Eolepidopterix* in the following characters: 1), the pronotum is short with a length of 0.05 mm and a width/length ratio of 8.0 (vs. pronotum relatively long with length of 0.19 mm and width/length ratio of 2.9); 2), the Sc vein is forked distally (vs. the Sc vein branching near the base of the forewing); and 3), the anterior margins of both the fore- and hind wings bear cilia (vs. lacking cilia). The wing of *Paleolepidopterix* is not well preserved. Based on the present specimen, *Petilicorpus* can be differentiated from *Paleolepidopterix* by its longer head and shorter pronotum. The venation of *Petilicorpus* resembles *Daiopterix* in the following characters: 1), the Sc and R_1_ are forked; and 2), the Rs_1+2_ stem is shorter than the Rs_3+4_ stem. In addition, *Petilicorpus* can be separated from *Daiopterix* by: 1), possession of a long Sc vein (vs. a short Sc vein); 2), the Sc_2_ vein extending beyond the Rs_1+2_ furcation (vs. the Sc_2_ not reaching the Rs_1+2_ furcation); and 3), the m crossvein is present (vs. m crossvein absent).

### *Petilicorpus cristatus* Zhang, Shih, Labandeira & Ren sp. nov. (Fig. 10)

urn:lsid:zoobank.org:act:7D597FB8-6196-4664-9BB6-087BCABF95B2.

#### Etymology

The specific name is derived from the Latin, *cristatus* (bearing a crest, cluster of plumes), in reference to setae on the head.

#### Type material

Holotype: CNU-LEP-NN-2012-007; ♀; left fore and hind wings well preserved; anal areas are obscured.

#### Locality and horizon

This specimen was collected from Daohugou Village, Shantou Township, Ningcheng County, Inner Mongolia Autonomous Region, China. The age is latest Middle Jurassic, near the Callovian–Oxfordian boundary.

#### Diagnosis

Same as generic diagnosis.

#### Description

**Figure 10:**
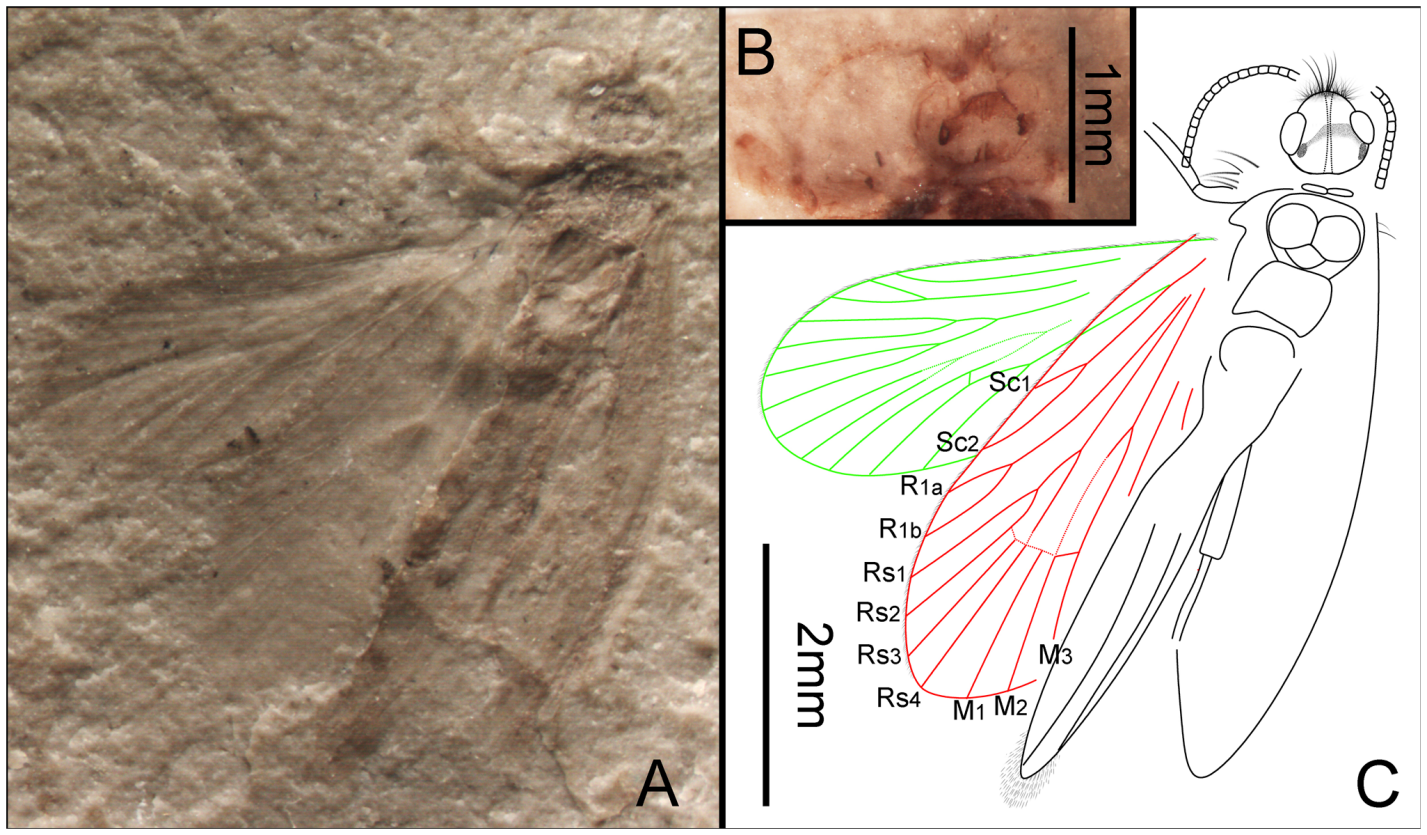
*Petilicorpus cristatus* gen. et sp. nov. Female, holotype, CNU-LEP-NN-2012-007. (A), Fossil specimen. (B), Head and associated structures. (C), Camera lucida drawing of (A), showing overall habitus.

Female. Body slender and entirely covered by setae. Head slightly longer than wide, anterior edge with clusters of setae pointing forward. Antennae filiform, less than half length of forewing; length of segments equal to their diameter. Eyes oval. A pair of chaetosemata present near eyes (Fig. 10B). Median ocellus absent. Prothorax much broader than length, with a pair of transverse structures that are adjacent mesially. Mesoscutum broader than long, posterior edge concave along midline; mesoscutellum flabellate, slightly shorter than mesoscutum. Mesothorax large. Long setae present on the sides of prothorax and mesothorax.

Anterior margins of both fore- and hind wings bear cilia. Forewing humeral vein indiscernible. Sc forked from distal 1/3 of the stem; Sc_1_ extended to the costal margin beyond the 1/3 the length of the wing from its base; Sc_2_ extended beyond the 1/2 length of the wing from its base. R_1_ forked, almost at same level as the Rs_1+2_ furcation; Rs 4-branched; Rs_4_ terminating at apex of forewing; Rs_1+2_ stalked ca. 0.4 of total vein length; Rs_3_ and Rs_4_ free; Rs_1+2_ stem slightly shorter than Rs_3+4_ stem. M 3-branched. Hyaline zones surround r–m crossvein and at Rs_3+4_ and M_1+2_ furcations. Crossvein m present, originating near M_1+2_ furcation and terminating at the midpoint of M_3_. Hind wing ca. 2.3 times as long as wide. Venation similar to forewing; Sc not forked, m crossvein absent and m_3_–cua_1_ crossvein present. CuA bifurcated. In female, apophyses slender, almost 2/3 as long as abdomen. Abdominal terminus (possibly the papillae anales) covered by dense long setae.

#### Measurements (in mm)

Body length 6.6; width 0.9. Forewing length 5.2; hind wing length 5.0, width ca. 2.1.

### *Longcapitalis* Zhang, Shih, Labandeira & Ren gen. nov

urn:lsid:zoobank.org:act:5DEA5662-B07C-4DF6-9784-008E56B7A61F.

#### Type species

*Longcapitalis excelsus* Zhang, Shih, Labandeira & Ren sp. nov.

#### Etymology

The generic name is derived from the Latin, *longus* (long, extended), and *capitalis* (head of a living creature), in reference to the elongate head of this species. The gender is masculine.

#### Distribution

Inner Mongolia Autonomous Region, China.

#### Diagnosis

Head longer than wide. Metatibiae lacking spines. Anterior margin of wings lacking cilia. Humeral vein in forewing present. Sc forked. R_1_ not forked. All furcations of Rs_1+2_, Rs_3+4_, and M_1+2_ almost at the same level with each other. Anal veins looping into a double-Y configuration. Hind wing with Sc and R_1_ not forked. Veins on hind wing lacking spines.

An Eolepidopterigidae affiliation is supported by: 1), wings homoneurous; 2), M 3-branched; and 3), crossvein absent between Sc and R.

#### Comparison

The venation of *Longcapitalis* resembles that of other Eolepidopterigidae, but this new genus lacks a R_1_ furcation in the forewing, making it different from all other species of Eolepidopterigidae. *Longcapitalis* shows great similarity to the genus *Undopterix*, but it differs from the latter in the following characters: 1), the R_1_ is not forked in both fore- and hind wings (vs. R_1_ forked in the fore- and hind wings); 2), the Rs_1+2_ stem is as long as the Rs_3+4_ stem (vs. the Rs_1+2_ stem shorter than the Rs_3+4_ stem); and 3), the anal veins form a double-Y loop (vs. the anal veins forming a single-Y loop). *Longcapitalis* differs from *Akainalepidopteron* by the following characters: 1), the R_1_ is not forked in both fore- and hind wings (vs. R_1_ forked); 2), the hind wing lacks spines (vs. spines present on several veins on hind wing); 3), the wings lack cilia (vs. cilia present on anterior margins of the fore- and hind wings); and 4), the metatibiae lack spines (vs. the metatibiae bearing irregularly arranged spines).

### *Longcapitalis excelsus* Zhang, Shih, Labandeira & Ren sp. nov. (Fig. 11)

urn:lsid:zoobank.org:act:4C76B3E1-673A-4D9C-958F-B866E4DD0055.

#### Etymology

The specific name is derived from the Latin, *excelsus* (tall, large), in reference to the comparatively large body size of this species.

**Figure 11:**
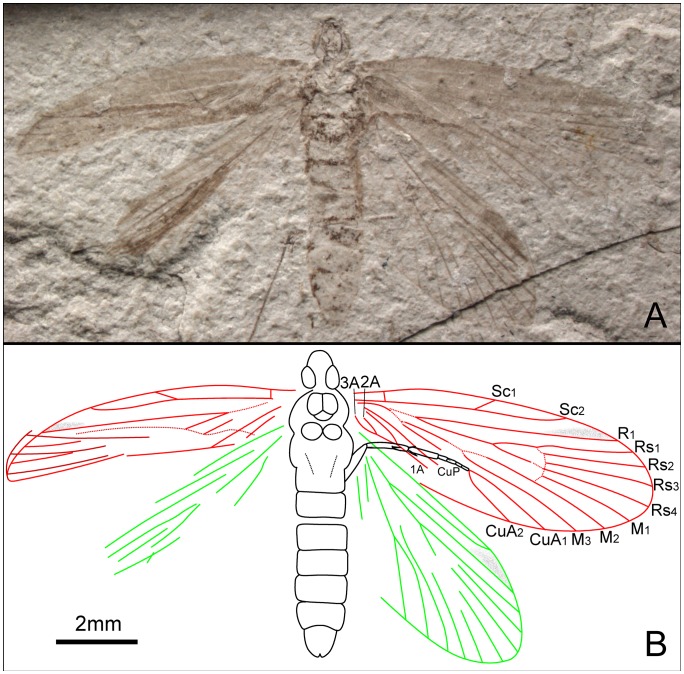
*Longcapitalis excelsus* gen. et sp. nov. Female, holotype, CNU-LEP-NN-2012-025P. (A), Fossil specimen. (B), Camera lucida drawing of (A), showing overall habitus.

#### Type material

Holotype: CNU-LEP-NN-2012-025P/C (part and counterpart); ♀; well preserved forewings, hindleg, and portion of hind wings.

#### Locality and horizon

This specimen was collected from Daohugou Village, Shantou Township, Ningcheng County, Inner Mongolia Autonomous Region, China. The age is latest Middle Jurassic, near the Callovian–Oxfordian boundary.

#### Diagnosis

Same as generic diagnosis.

#### Description

Head relatively long (ca. 0.9 mm), longer than wide; compound eyes elongate oval. Mesonotum slightly broader than long, with median suture; posterior edge concave along midline; mesoscutellum small, ca. half length of mesoscutum. Metafemora with a spine at the end of femora. Metatibiae with one pair medial spurs and one pair apical spurs. Metatarsi 5-segmented, all tarsal segments with terminal spinules.

Forewing 2.7 times as long as wide, bearing a humeral vein; pterostigma present. Sc forked, at distal 2/5 of the stem, Sc_1_, almost extending to the midpoint of wing costal margin; Sc_2_ extending to costal margin of wing, beyond 2/3 length of wing from base. R_1_ not forked; Rs 4-branched; Rs_4_ extending to apex of forewing; Rs_1+2_ stalked ca. to 0.4 of the total vein length; Rs_3_ and Rs_4_ free; and Rs_1+2_ stem subequal to Rs_3+4_ stem. M 3-branched. Hyaline zones surround r–m crossvein at Rs_1+2_ furcation, and at the Rs_3+4_, M_1+2_ and M_1+2_–M_3_ furcations. CuA bifurcated; CuP simple. Anal veins looping into a double-Y configuration. Hind wing 2.6 times as long as wide. Sc not forked. R_1_ not forked. Rs 4-branched. M 3-branched. CuA bifurcated; CuP simple. Anal area not well preserved.

#### Measurements (in mm)

Body length ca. 7.9; width 1.7. Forewing length 8.2; width 3.0. Hind wing length ca. 7.3; width 2.8.

### *Grammikolepidopteron* Zhang, Shih, Labandeira & Ren gen. nov

urn:lsid:zoobank.org:act:6EB3F8BB-B784-4239-8FEE-BEA4B8FA5D9D.

#### Type species

*Grammikolepidopteron extensus* Zhang, Shih, Labandeira & Ren sp. nov.

#### Etymology

The generic name is from the Greek, combining *grammikos* (line, linear), in reference to the linearly aligned branching points of veins R_1_ and Rs_1_ to Rs_3_; and *lepidos*, meaning “scale” or “flake,” representing the Lepidoptera, the ordinal assignment of this species; and *pteron*, meaning “wing” or “fin.”. The gender is masculine.

#### Distribution

Inner Mongolia Autonomous Region, China.

#### Diagnosis

Body small, less than 4.0 mm. Forewing elongate. Humeral vein and sc–r crossvein absent. Sc and R_1_ not forked. Rs_3_ terminating only slightly before wing apex. Branching points of R_1_ and Rs_1_ to Rs_3_ linearly aligned. M divided into M_3_ and M_1+2_. M_1+2_ subdivided iteratively into veins M_1_ and M_2_ at an angle of ca. 15 degrees; M_1_ linear and smooth. CuA bifurcated; CuP simple. Three anal veins looping into a double-Y configuration. Apophyses short.

An Eolepidopterigidae affiliation is supported by: 1), antennae less than half the length of forewing; 2), M 3-branched; and 3), crossvein absent between Sc and R.

#### Comparison

*Grammikolepidopteron* resembles *Netoxena* Sohn, 2012, but *Grammikopidopteron* is separated from *Netoxena* by: 1), absence of a sc–r crossvein (vs. sc–r crossvein present); 2), the M_l+2_ furcation extending beyond the CuA furcation (vs. the CuA furcation extending beyond the M_l+2_ furcation); and 3), three anal veins looping into a double-Y configuration (vs. two anal veins looping into a single-Y configuration).

*Grammikolepidopteron* resembles one fossil species within *Micropterix*, *Micropterix pervetus* Cockerell, 1919 [42]. However, *Grammikolepidopteron* differs from *M*. *pervetus* in: 1), the forewing having Sc and R_1_ veins that are not forked (vs. Sc and R_1_ that are forked); 2), anal veins that form a double-Y configuration (vs. 1A and 2A veins that are parallel); and 3), presence of a CuP vein (vs. absence of the CuP vein).

### *Grammikolepidopteron extensus* Zhang, Shih, Labandeira & Ren sp. nov. (Fig. 12)

urn:lsid:zoobank.org:act:AD3B8CA4-5935-48A5-823F-46C48D0D2C01.

#### Etymology

The specific name is derived from the Latin, *extensus* (outstretched, extended), in reference to the expansive forewings.

**Figure 12:**
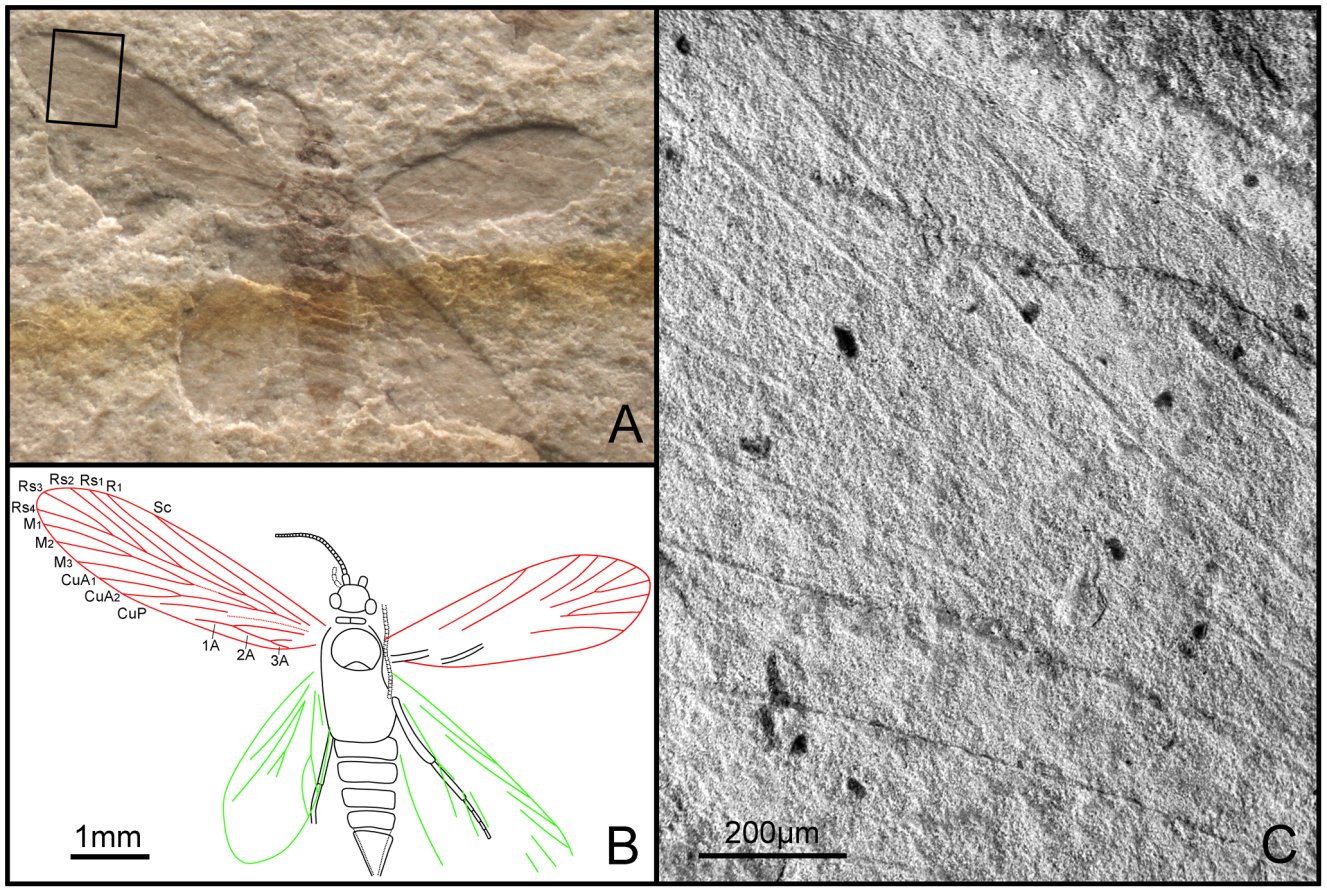
*Grammikolepidopteron extensus* gen. et sp. nov. Female, holotype, CNU-LEP-NN-2012-006P. (A), Fossil specimen. (B), Camera lucida drawing of (A), showing overall habitus. (C), SEM micrograph showing the left forewing venation, enlarged from the rectangular template in (A).

#### Type material

Holotype: CNU-LEP-NN-2012-006P/C (part and counterpart); ♀; right forewings well preserved; body and hind wing not well preserved. Forewings asymmetrical; left forewing and right hind wing are much longer and slender than the right forewing and left hind wing. This asymmetry probably is attributable to sediment deformation.

#### Locality and horizon

This specimen was collected from Daohugou Village, Shantou Township, Ningcheng County, Inner Mongolia Autonomous Region, China. The age is latest Middle Jurassic, near the Callovian–Oxfordian boundary.

#### Diagnosis

Same as generic diagnosis.

#### Description

Antennae with scape swollen; flagellum filiform; length of segments equal to their diameter. Eyes relatively small. Last segment of a maxillary (or labial) palpus visible. Posterior edge of mesoscutum concave; mesoscutellum small, half the length of mesoscutum. Metatarsi 5-segmented, tarsomere I longest, almost subequal to the total length of tarsomeres II, III, IV and V combined. Female genitalia with long ovipositor partially preserved.

Forewings distinctly narrow and elongate, slightly rounded apically, ca. 3.6 times as long as wide. Sc not forked, extending to costal margin of wing, at 2/3 the length of wing from base. R_1_ not forked, extending to costal margin of wing at a point 4/5 length of wing from base; Rs 4-branched; Rs_3_ terminating slightly before wing apex; branching points of R_1_ and Rs_1_ to Rs_3_ linearly aligned. M arising from CuA, 3-branched; M_1_ and M_2_ stalked, ca. 0.4 of their total length. CuA furcation distal of M furcation and before the M_1+2_ furcation; CuP simple. Three anal veins looping into a double-Y configuration. Hind wing relatively elongate, rounded apically, 3 times as long as wide. Sc and R_1_ not forked. CuA bifurcated.

#### Measurements (in mm)

Body length over 3.8, as preserved (missing part of terminalia); width 0.9. Forewing length ca. 4.2 (left wing) and 3.5 (right wing); width ca. 1.1 (left wing) and 1.2 (right wing). Hind wing length ca. 2.9 (left wing) and 3.3 (right wing); width ca. 1.2 (left wing) and 1.3 (right wing).

### Suborder *Incertae sedis*

### Family Mesokristenseniidae Huang, Nel & Minet, 2010

#### Type genus

*Mesokristensenia* Huang, Nel & Minet, 2010 [25].

#### Distribution

Inner Mongolia Autonomous Region, China.

#### Emended diagnosis

Antenna less than half length of forewing. Labial palpi long. Mesotibia lacking medial spurs; spur formula of legs: 1-1-4. Number of forewing vein branches: Sc-2, R_1_-1, Rs-4, M-4, CuA-2, CuP-1, A-3; Rs_3_ and Rs_4_ separate. Ovipositor nonpiercing, with anterior apophyses.

The synapomorphies for the Mesokristenseniidae are mesotibia with only an apical spur, wings homoneurous, R_1_ unforked, M vein 4-branched, and ovipositor with anterior apophyses.

The lepidopteran affiliation of this family has been established by Huang *et al*. [25] based on the following characters: 1), apex of the foretibia with at most one spur; 2), wings covered with overlapping scales; 3), wings lacking nygmata; and 4), the M_1_ sharply angulate at the junction with r–m crossvein.

#### Genera included

*Kladolepidopteron* gen. nov. and *Mesokristensenia* Huang, Nel & Minet, 2010.

### *Mesokristensenia* Huang, Nel & Minet, 2010

#### Type species

Mesokristensenia latipenna Huang, Nel & Minet, 2010.

#### Distribution

Inner Mongolia Autonomous Region, China.

#### Emended diagnosis

Antenna short, ca. 1/3 the length of forewing; scape prominently swollen; pedicel relatively robust. Metatibia with spines and two pairs of spurs. M_1_, after separation from M_2_, subtended by an angle of greater than 60 degrees, sharply angulate at junction with r–m crossvein. Crossvein sc–r absent.

#### Species included

*M. angustipenna* Huang, Nel & Minet, 2010 [25]; *M. latipenna* Huang, Nel & Minet, 2010 [25]; *M. sinica* Huang, Nel & Minet, 2010 [25] and *M. trichophora* sp. nov.

### *Mesokristensenia sinica* Huang, Nel & Minet, 2010 (Figs. 1F and 13)

#### Type materials

Holotype NIGP-150462 deposited in Nanjing Institute of Geology and Palaeontology [25]. Additional specimen CNU-LEP-NN-2009-003; ♂; forewings well preserved anal areas are covered by hind wings.

**Figure 13:**
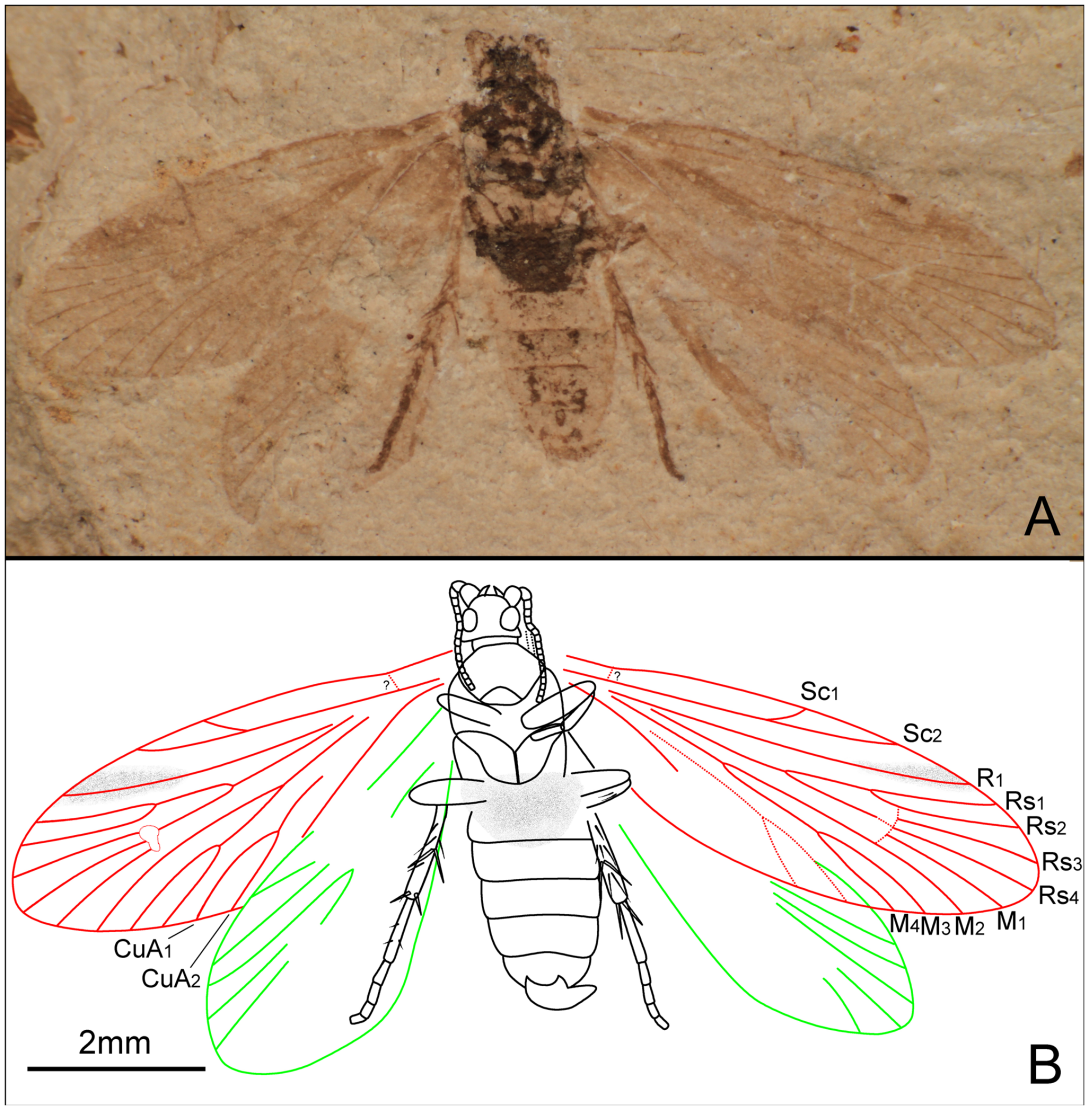
*Mesokristensenia sinica* Huang, Nel & Minet. Male, CNU-LEP-NN-2009-003. (A), Fossil specimen. (B), Camera lucida drawing of (A), showing overall habitus.

#### Locality and horizon

This specimen was collected from Daohugou Village, Shantou Township, Ningcheng County, Inner Mongolia Autonomous Region, China. The age is latest Middle Jurassic, near the Callovian–Oxfordian boundary.

#### Description of additional specimen

Antenna with the scape prominently swollen and pedicel relatively large; flagellum filiform; length of segments equal to their diameter. Compound eyes moderately developed. Apex of labial palpi acute. Mesothorax large; mesonotum slightly broader than long, posterior edge concave; mesoscutellum small, half the length of mesoscutum. Mesofemora and metafemora robust. Metatibia with irregularly arranged spines; a pair of apical spurs and additional spurs arising from distal third or fourth of tibia; all spurs ca. 2 times diameter of tibia. Metatarsi 5-segmented; tarsomere I longest, with short spine.

Forewing 3.2 times as long as wide; prominent pterostigma present. Humeral vein possibly present. Sc forked, from distal 1/3 of the stem; Sc_2_ extending to costal margin of wing at 2/3 length from wing base; sc–r crossvein absent. R_1_ not forked; Rs 4-branched; Rs_4_ slightly below the apex of forewing; Rs_1+2_ stalked ca. 0.3 of their total length. M 4-branched, stem M_1+2_ longer than stem M_3+4_. Hyaline zones surrounding r–m crossvein at Rs_3+4_ and M_1+2_ furcations. CuA forked before M_3+4_ furcation. Hind wing 2.45 times as long as wide.

#### Measurements (in mm)

CNU-LEP-NN-2009-003: body length ca. 5.0; width 1.5. Forewing length ca. 6.0; width 1.9. Hind wing length 4.9; width 2.0.

#### Remarks

This specimen, collected from the same locality as the holotype of *M. sinica*, matches the characters of *M. sinica* in the venation, wing index and body size. We deem this specimen conspecific with *M. sinica*.

### *Mesokristensenia trichophora* Zhang, Shih, Labandeira & Ren sp. nov. (Fig. 14)

urn:lsid:zoobank.org:act:A70E3B9C-22EC-455E-8202-78153C4B8E90.

#### Etymology

The specific name is derived from the Greek, *trichos* (hair), and *phoro*– (bearing, carrying), in reference to hirsute antennal scape present in this species.

#### Type material

Holotype: CNU-LEP-NN-2012-032; ♀; wings well preserved; legs partially preserved.

#### Locality and horizon

This specimen was collected from Daohugou Village, Shantou Township, Ningcheng County, Inner Mongolia Autonomous Region, China. The age is latest Middle Jurassic, near the Callovian–Oxfordian boundary.

#### Diagnosis

Antennal scape with setae. Forewing of *M. trichophora* (with wing index of 0.34) narrower than *M. latipenna* (wing index 0.38); slightly broader than *M. sinica* (wing index 0.32/0.33), and broader than *M. angustipenna* (wing index 0.31).

#### Description

**Figure 14:**
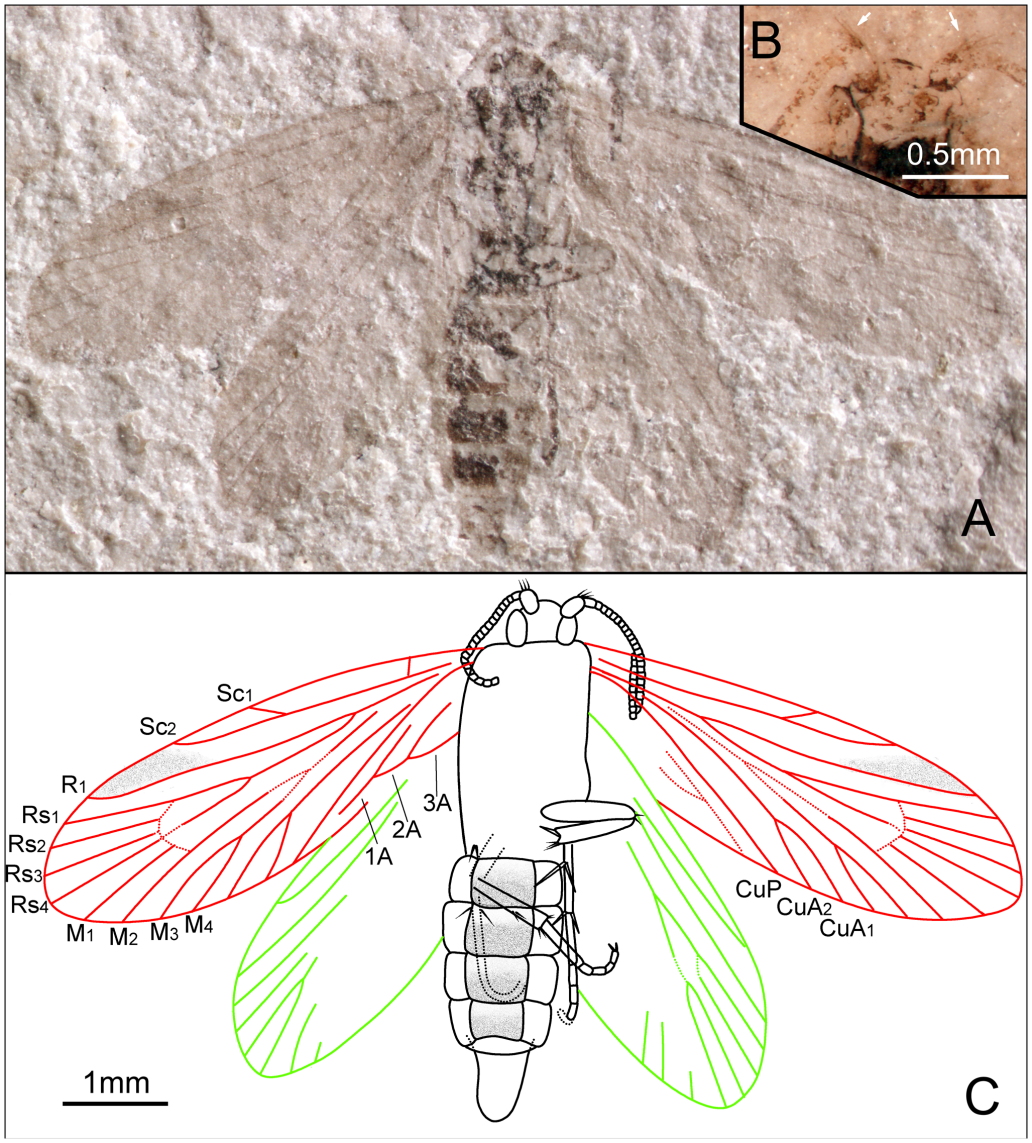
*Mesokristensenia trichophora* sp. nov. Female, holotype, CNU-LEP-NN-2012-032. (A), Fossil specimen. (B), Head, showing scape with setae distally. (C), Camera lucida drawing of (A), showing overall habitus.

Compound eyes oval; antennae filiform; scape large and elongate, with setae distally placed (Fig. 14B); pedicel longer than one segment of flagellum; flagellum slender, the length of segments basally ca. 0.5 times as long as their diameter; length equal to their diameter distally. Metatibia with one pair of medial spurs, ca. 3 times as long as the diameter of the tibia; one pair of apical spurs, ca. 1.5 times as long as the diameter of the tibia; metatarsi 5-segmented, tarsomere I longest. Ovipositor relatively long and robust.

Forewing 2.9 times as long as wide; prominent pterostigma present. Forewing bearing a humeral vein. Sc forked at distal 1/3 of the stem; Sc_2_ extending to costal margin of wing at 2/3 distance from wing base; sc–r crossvein absent. R_1_ not forked; Rs 4-branched; Rs_4_ extending to the apex of forewing; Rs_1+2_ stalked ca. 1/3 of their total length; Rs_3_ and Rs_4_ free. M 4-branched; M_1+2_ stem longer than M_3+4_ stem. Hyaline zones surround r–m crossvein at Rs_1+2_, Rs_3+4_ and M_1+2_ furcations. CuA forked before M_3+4_ furcation; CuA_1+2_ stalked over half of their length; CuP simple. Three anal veins looping into a double-Y configuration; 1A and 2A separate beyond basal half of vein length. Hind wing length ca. 2.6 times the width. Sc not forked; R_1_ not forked; Rs 4-branched; Rs_3+4_ stem slightly longer than stem Rs_1+2_.

#### Comparison

*M. trichophora* differs from other species of *Mesokristensenia* in bearing an antennal scape with setae and in the afore-mentioned wing index.

#### Measurements (in mm)

Body length ca. 5.1; width 1.3. Forewing length 5.0; width 1.7. Hind wing length 4.1; width 1.6.

### *Kladolepidopteron* Zhang, Shih, Labandeira & Ren gen. nov

urn:lsid:zoobank.org:act:3D602334-A178-4752-8EB0-03053E2982C7.

#### Type species

*Kladolepidopteron oviformis* Zhang, Shih, Labandeira & Ren sp. nov.

#### Etymology

The generic name is derived from the Greek, combining *klados* (twig, branch, stem), in reference to the presence of crossvein sc–r; and *lepidos*, meaning “scale” or “flake,” also signifying assignment to the order Lepidoptera; and *pteron*, the word for “wing” or “fin.” The gender is masculine.

#### Distribution

Inner Mongolia Autonomous Region, China.

#### Diagnosis

Vein M 4-branched in forewing. Prothorax with a pair of oval structures. Metatibia lacking spines. Crossvein sc–r present at forewing base.

A Mesokristenseniidae affiliation is supported by: 1), antenna less than half length of forewing; 2), mesotibia with only one apical spur; 3), wings homoneurous; 4), R_1_ unforked; and 5), M 4-branched.

#### Comparison

*Kladolepidopteron* differs from *Mesokristensenia* in: 1), having the sc–r crossvein present at the base of the forewing (vs. sc–r absence); and 2), the metatibia lacking spines (vs. hind legs bearing irregularly arranged spines).

Compared to the sole genus of the Agathiphagidae, *Agathiphaga*, *Kladolepidopteron* can be distinguished by the following characters: 1), the sc–r crossvein is present at the base of the forewing (vs. the sc–r crossvein present near the distal tip of the Sc_2_); 2), absence of the m–cup and cup–a crossveins (vs. presence of the m–cup and cup–a crossveins); and 3), presence of the m crossvein (vs. absence of the m crossvein).

#### Species included

Kladolepidopteron oviformis sp. nov; K. parva sp. nov.; and K. subaequalis sp. nov.

### *Kladolepidopteron oviformis* Zhang, Shih, Labandeira & Ren sp. nov. (Fig. 15)

urn:lsid:zoobank.org:act:5FAB0FF0-53B9-4117-8363-FA375B4909B9.

#### Etymology

The specific name is derived from the Latin *ovum* (egg, egg-like); and *formis*, (shape of, likeness, form), in reference to the pair of oval structures on the prothorax.

#### Type material

Holotype: CNU-LEP-NN-2009-007P/C (part and counterpart); ♀; well preserved body and left forewing; mid and hind legs partially preserved.

#### Locality and horizon

This specimen was collected from Daohugou Village, Shantou Township, Ningcheng County, Inner Mongolia Autonomous Region, China. The age is latest Middle Jurassic, near the Callovian–Oxfordian boundary.

#### Diagnosis

Body relatively large. Wing index 0.33. Forewing with crossvein m present. In the hind wing, Rs_1+2_ stem shorter than Rs_3+4_ stem.

#### Description

Body slender. Compound eyes rounded. Antenna filiform, slender, length of segments 1.5 times their diameter.

**Figure 15:**
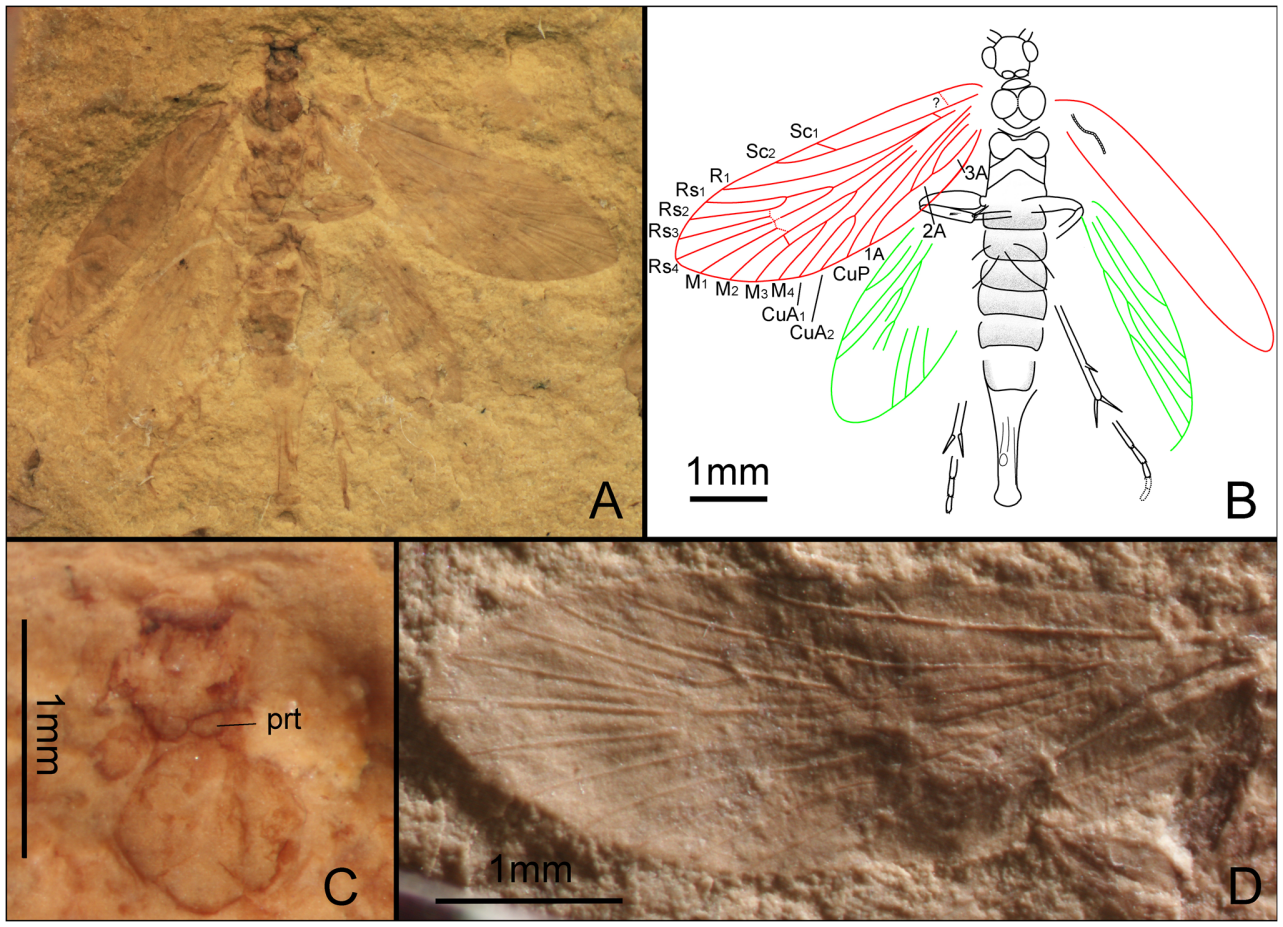
*Kladolepidopteron oviformis* gen. et sp. nov. Female, holotype, CNU-LEP-NN-2009-007C. (A), Fossil specimen. (B), Camera lucida drawing of (A), showing overall habitus. (C), Head and thorax. (D), Forewing. Abbreviation: prt, prothorax.

Prothorax relatively large, with a pair of oval, slightly transverse structures that are adjacent mesially (Fig. 15C). Mesothorax with relatively large prescutum; mesoscutum broader than long; mesoscutellum small, posterior edge concave along midline, slightly shorter than mesoscutum. Mesoscutellum fan-shaped; metascutum dumbbell shaped; metascutellum relatively large and broad. Mesofemora ca. 2 times as wide as mesotibia; mesofemora and mesotibia almost subequal in length. Mesotibia with one apical spur; two spines present at middle of mesotibia. Metatibia with one pair of medial spurs, one pair slightly longer than diameter of tibia, and other pair of apical spurs ca. 2 times as long as the diameter of the tibia. Metatarsi 5-segmented; two very short setae present on the apex of each tarsomere.

Forewings moderately slender, gradually tapering to a subacute apex (Fig. 15D). Length ca. 3.0 times the width. Forewing bearing a humeral vein. Sc forked, from distal 1/4 of the stem; Sc_2_ extending to costal margin of wing at 3/5 length of wing from base. Crossvein sc–r present. R_1_ not forked; Rs 4-branched; Rs_4_ extending to the apex of forewing; Rs_1_ and Rs_2_ stalked; Rs_1+2_ stalked ca. 0.38 of their total length; Rs_3_ and Rs_4_ free. M 4-branched; stem M_1+2_ longer than stem M_3+4_, M_1_ after separation from M_2_ subtending an angle of 60 degrees, sharply angulate at junction with crossvein r–m. Crossvein m–ca. originating near M_1+2_ furcation and terminating near the midpoint of M_3_. Hyaline zones surrounding r–m crossvein at Rs_3+4_ and M_1+2_ furcations. CuA_1+2_ stalked over half of their length; CuP simple. Three anal veins looping into a double-Y configuration; 1A and 2A separate at basal half. Hind wing length ca. 2.8 times the width. Sc not forked. R_1_ not forked; Rs 4-branched; Rs_3+4_ stem ca. 2 times as long as stem Rs_1+2_. Abdomen lacking specialized integumental modifications. Ovipositor long; apophyses not evident.

#### Measurements (in mm)

Body length ca. 6.3; width 1.1. Forewing length 4.8; width 1.6. Hind wing length ca. 3.6; width 1.3.

### *Kladolepidopteron subaequalis* Zhang, Shih, Labandeira & Ren sp. nov. (Fig. 16)

urn:lsid:zoobank.org:act:4356A7DD-DEF5-450C-B17B-389E685ABA09.

#### Etymology

The specific name is derived frpm the Latin preposition, *sub* (under, below), and *aequalis* (equal), in reference to the subequal length of the basal stems to the hind wing veins Rs_1+2_ and Rs_3+4_.

#### Type materials

Holotype: CNU-LEP-NN-2012-020; sex unknown; well preserved forewings and left hind wing. Paratype. CNU-LEP-NN-2012-022; sex unknown; only forewing, with distal part not preserved.

#### Diagnosis

Wing index 0.32. In forewing, crossvein m absent. Hind wing relatively broad; the stem Rs_1+2_ and Rs_3+4_ subequal in length.

#### Comparison

*Kladolepidopteron. subaequalis* resembles *K. oviformis* in: 1), the forewing index (0.32 vs. 0.33); and 2), the wing apex is moderately acute. *Kladolepidopteron subaequalis* differs from *K. oviformis* in the following characters: 1), the crossvein m absent (vs. crossvein m present); 2), the hind wing has a slightly shorter Rs_1+2_ stem than the Rs_3+4_ stem (vs. Rs_1+2_ stem only half length of Rs_3+4_ stem); and 3), the hind wing of *K. subaequalis* is broader than that of *K. oviformis*.

#### Description

Mesothorax large, with prominent scutum and small scutellum; prescutum not preserved. Mesoscutum with distinct median suture, broader than long, posterior edge concave along midline; mesoscutellum fan-shaped, half the length of mesoscutum; metascutum dumbbell shaped.

**Figure 16:**
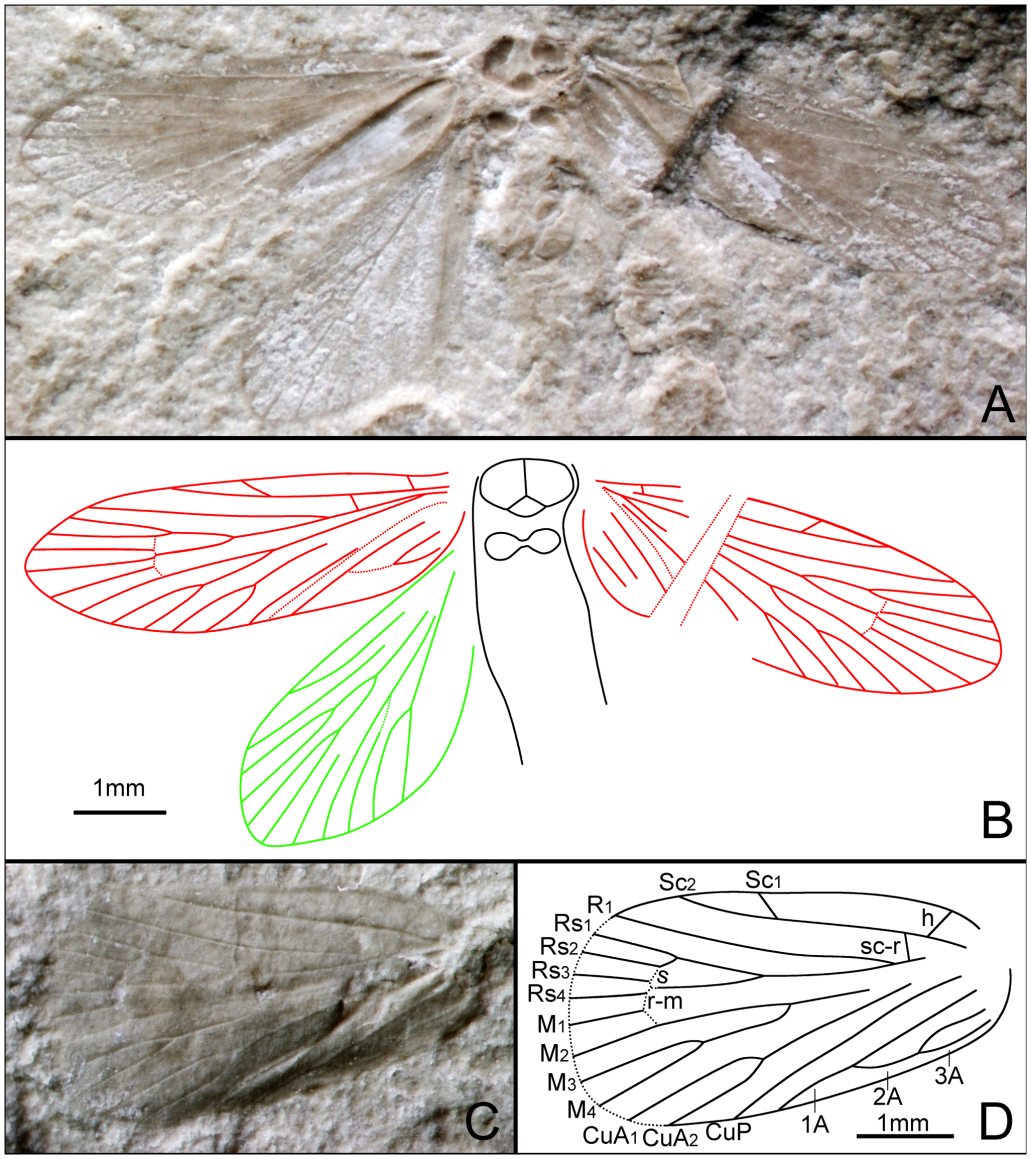
*Kladolepidopteron subaequalis* gen. et sp. nov. Holotype, CNU-LEP-NN-2012-020. (A), Fossil specimen. (B), Camera lucida drawing of (A), showing overall habitus. Paratype, CNU-LEP-NN-2012-022. (C), Fossil specimen. (D), Camera lucida drawing of wing at (C).

Forewing moderately slender, tapering gradually to apex. Length ca. 3.1 times the width. Forewing bearing a humeral vein. Sc forked from distal 1/3 of the stem; Sc_2_ extending to costal margin of wing at 3/5 length of wing from base. Crossvein sc–r present. R_1_ not forked; Rs 4-branched; Rs_4_ extending to the apex of forewing; Rs_1_ and Rs_2_ stalked, Rs_1+2_ stalked ca. 0.35 of their total length; Rs_3_ and Rs_4_ free; Rs_1+2_ furcation almost at same level as Rs_3+4_. Crossvein s present (Figs. 16C, D). M 4-branched; M_1_, after separation from M_2_ subtending an angle of 75–80 degrees, sharply angulate at junction with r–m crossvein (Figs. 16C, D); stem M_1+2_ longer than stem M_3+4_. CuA bifurcated; CuP simple. Three anal veins forming a double-Y configuration; 1A and 2A separate at basal half. Hind wing length ca. 2.3 times the width; broader than forewing. Sc not forked. R_1_ not forked; Rs 4-branched; Rs branching into veins Rs_1+2_ and Rs_3+4_, and then further subdividing into four branches of Rs_1_, Rs_2_, Rs_3_, and Rs_4_; furcations of Rs_1+2_, and Rs_3+4_ almost at the same level to each other. M 4-branched. CuA bifurcated; CuP difficult to discern.

#### Measurements (in mm)

Forewing length 5.0; width 1.6. Hind wing length ca. 4.1; width 1.8.

### *Kladolepidopteron parva* Zhang, Shih, Labandeira & Ren sp. nov. (Fig. 17)

urn:lsid:zoobank.org:act:032CD405-AC08-4D0B-A489-FB6231FE7560.

#### Etymology

The specific name is derived from the Latin *parva* (small, little, stunted), in reference to its small body size.

#### Type material

Holotype: CNU-LEP-NN-2012-011P/C (part and counterpart); ♂; well preserved forewing and hind wing; anal wing region indiscernible.

#### Locality and horizon

This specimen was collected from Daohugou Village, Shantou Township, Ningcheng County, Inner Mongolia Autonomous Region, China. The age is latest Middle Jurassic, near the Callovian–Oxfordian boundary.

#### Diagnosis

The new species has narrower wings (wing index 0.31) than *K. oviformis* (wing index 0.33) and *K. subaequalis* (wing index 0.32). Crossvein m absent in forewing. Hind wing with Rs_1+2_ forking almost at the same level as Rs_3+4_.

#### Comparison

*Kladolepidopteron parva* resembles *K. oviformis* and *K. subaequalis* in venation. However, *K. parva* differs from *K. oviformis* by: 1), its short body, ca. 4.1 mm in length (vs. a relatively long body ca. 6.3 mm long); 2), short forewing length of 4.1 mm (vs. long forewing length of 4.8 mm); and 3), the Rs_1+2_ stem slightly shorter than the Rs_3+4_ stem (vs. the Rs_1+2_ stem only half the length of the Rs_3+4_ stem). *Kladolepidopteron parva* differs from *K. subaequalis* by: 1), its short forewing (vs. long forewing with a length of 5.0 mm); and 2), a forewing without a pterostigma (vs. an otherwise prominent pterostigma).

#### Description

**Figure 17:**
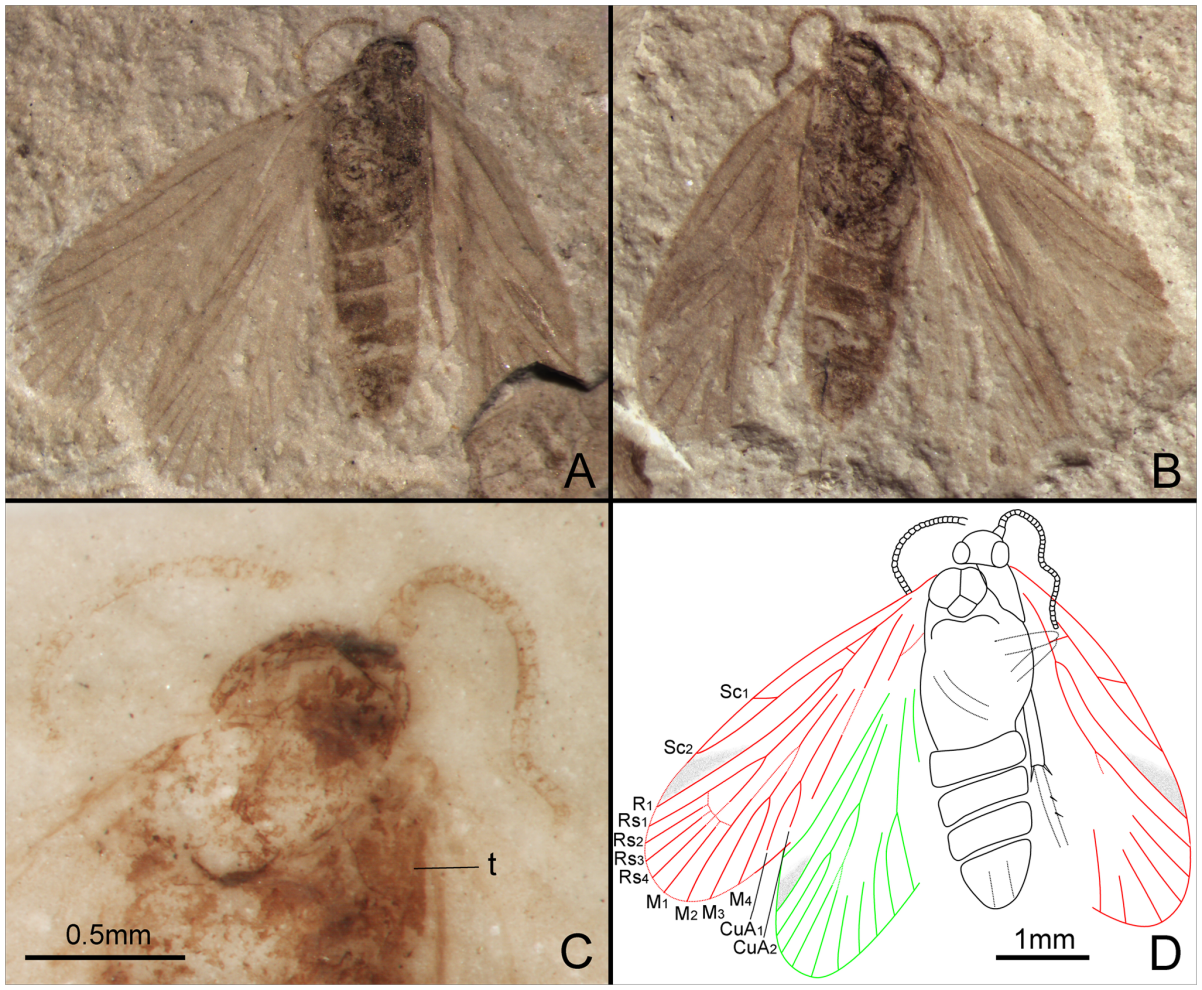
*Kladolepidopteron parva* gen. et sp. nov. Male, holotype, CNU-LEP-NN-2012-011P/C. (A), Fossil specimen (counterpart). (B), Fossil specimen (part). (C), Head and thorax. (D), Camera lucida drawing of (A), showing overall habitus. Abbreviation: t, tegula.

Antenna filiform, ca. half the length of forewing; scape swollen; flagellum filiform; length of segments equal to their diameter. Compound eyes widely separated. Tegula large, triangular (Fig. 17C). Mesoscutum with median suture, slightly broader than long; posterior edge of mesoscutum concave along midline. Mesoscutellum small, fan-shaped, slightly shorter than mesoscutum. Metatibia with a pair apical spurs, ca. 1.5 times the diameter of the tibia; medial spurs difficult to discern.

Forewing ca. 3.2 times as long as wide; a prominent pterostigma present. Forewing lacking a humeral vein. Sc forked from distal 1/3 of the stem; Sc_2_ extending to costal margin of wing, beyond 2/3 the length of wing from base. Crossvein sc–r present; R_1_ not forked; Rs 4-branched; Rs_1+2_ stalked ca. 1/2 of vein total length; Rs_3_ and Rs_4_ free. M 4-branched. Hyaline zones surrounding r–m crossvein at Rs_1+2_, Rs_3+4_, M_1+2_, and M_1+2_–M_3_ furcations and along CuP. CuA bifurcated; CuP simple. Hind wing Sc not forked. R_1_ not forked; Rs 4-branched; Rs_1_ and Rs_2_ stalked; Rs_3_ and Rs_4_ stalked. M 3-branched. CuA bifurcated.

#### Measurements (in mm)

Body length ca. 4.1; width 1.2. Forewing length ca. 4.1; width ca. 1.3. Hind wing length ca. 3.4.

### Family Ascololepidopterigidae Zhang, Shih, Labandeira & Ren fam. nov

urn:lsid:zoobank.org:act:4C3FD7D0-DD02-4575-BA4A-94600DFD80EB.

#### Type genus

*Ascololepidopterix* Zhang, Shih, Labandeira & Ren gen. nov.

#### Distribution

Inner Mongolia Autonomous Region, China.

#### Diagnosis

Antennae filiform, ca. half the length of forewing. Metatibiae lacking medial spurs, at most with one pair of short apical spurs. Both pairs of wings homoneurous. In forewing, number of vein branches as follows: Sc-1/2, R_1_-2, Rs-4, M-4, CuA-2, CuP-1, A-3, and furcations of Rs_1+2_, and Rs_3+4_ almost at the same level of branching with each other. Crossveins m–cua and cua–cup present. Ovipositor with apophyses anteriores.

The synapomorphies for the Ascololepidopterigidae fam. nov. are: 1), metatibiae lacking medial spurs; 2), wings homoneurous; 3), forewing R_1_ forked; 4), M vein 4-branched; 5), crossveins m–cua and cua–cup present; and 6), ovipositor with apophyses anteriores.

This family is defined as a clade of the Amphiesmenoptera by having a double anal furcation on the forewing and a hind wing M vein with at most three branches. This family is assigned to Lepidoptera by presence of a M_1_ vein which, after separation from M_2_ vein, subtends an angle greater than 60 degrees and is sharply angulate at the junction with the r–m crossvein.

#### Comparison

This new family with a 4-branched M is different from most other Lepidoptera, except for Agathiphagidae and Mesokristenseniidae. The differences among these three families are listed in Table 2.

**Table 2:** Character comparisons of Agathiphagidae, Mesokristenseniidae and Ascololepidopterigidae.

Character	Family		
	Agathiphagidae	Mesokristenseniidae	Ascololepidopterigidae fam. nov.
**Spur formula**	1-4-4	1-1-4	metatibia without medial spur; foretibia and mesotibia unknown
**Subcostal vein (Sc)**	forked	not forked or forked	not forked or forked
**Radial vein (R_1_)**	not forked	not forked	Forked
**Furcations of Rs_1+2_ and Rs_3+4_**	Furcation of Rs_3+4_ distally, distinctly beyond furcation of Rs_1+2_	approximately at the same level to each other	approximately at the same level to each other
**Crossvein m–cua**	Present	absent	Present
**Crossvein cua–cup**	Present	absent	Present

#### Genera included

*Trionolepidopteron* gen. nov., *Pegolepidopteron* gen. nov. and *Ascololepidopterix* gen. nov.

Key to known genera of Ascololepidopterigidae fam. nov**.**

**1**. Forewing with Sc not forked …………………………………  .………………….…………………*Ascololepidopterix* gen. nov.

**–** Forewing with Sc forked…………………………………….**2**

**2**. Forewing length more than 7.0 mm; forewing

 lacking crossvein rs_1_–rs_2_; crossvein r present …………  ………………………...…………….*Pegolepidopteron* gen. nov.

**–** Forewing length less than 5.0 mm; forewing

 with crossvein rs_1_–rs_2_; crossvein r absent…………………  ……………….……………………*Trionolepidopteron* gen. nov.

### *Ascololepidopterix* Zhang, Shih, Labandeira & Ren gen. nov

urn:lsid:zoobank.org:act:8F418F30-2094-4D03-A8C1-455119AE64EC.

#### Type species

*Ascololepidopterix multinerve* Zhang, Shih, Labandeira & Ren sp. nov.

#### Etymology

The generic name is derived from the Greek, *a-*,meaning “not” or “without”; and *scolos*, meaning a “spur”, “thorn” or “anything pointed”, referring to the absence or reduction of metatibial spurs in this Middle Jurassic fossil; and *lepidos*, meaning “scale” or “flake,” and the ordinal name of Lepidoptera, to which the fossil belongs; and *pteron*, meaning “wing” or “fin.” The gender is feminine.

#### Distribution

Inner Mongolia Autonomous Region, China.

#### Diagnosis

Body relatively large. Forewing Sc not forked. R_1_ forked. Crossveins s and r–m present. Stem M_1+2_ shorter than stem M_3+4_; crossvein m present, originating near M_1+2_ furcation and terminating on M_3+4_; crossvein m_3+4_–cua_1_, distant from the M and CuA furcations. Crossvein cua–cup originating at ca. 2/3 the length of CuA from its base and terminating beyond the midpoint of CuP. Hind wing R_1_ not forked. The distal part of 2A curved upward.

An Ascololepidopterigidae affiliation is supported by: 1), wings homoneurous; 2), R_1_ forked; 3), M 4-branched; and 4), crossveins m–cua and cua–cup present.

### *Ascololepidopterix multinerve* Zhang, Shih, Labandeira & Ren sp. nov. (Fig. 18)

urn:lsid:zoobank.org:act:7B3EA809-9A7A-4AE9-AD90-7075315CCB91.

#### Etymology

The specific name is derived from the Latin, *multi* (many, much), and *nervis* (sinew, tendon, vein), in reference to the numerous crossveins in the forewings.

**Figure 18:**
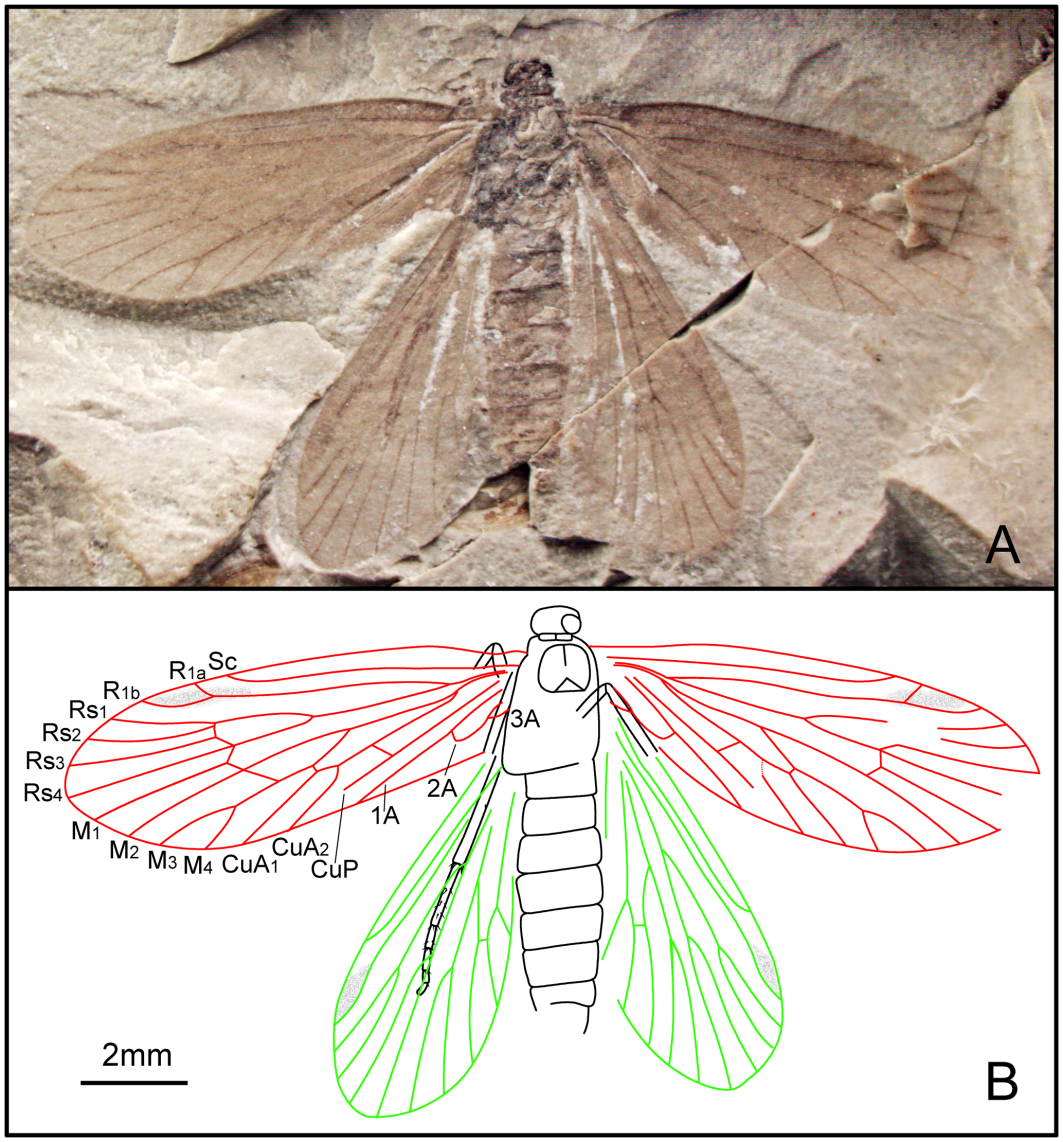
*Ascololepidopterix multinerve* gen. et sp. nov. Holotype, CNU-LEP-NN-2012-028. (A), Fossil specimen. (B), Camera lucida drawing of (A), showing overall habitus.

#### Type material

Holotype: CNU-LEP-NN-2012-028; sex unknown; well preserved forewings and hind wings; hind legs partially preserved.

#### Locality and horizon

This specimen was collected from Daohugou Village, Shantou Township, Ningcheng County, Inner Mongolia Autonomous Region, China. The age is latest Middle Jurassic, near the Callovian–Oxfordian boundary.

#### Diagnosis

Same as generic diagnosis.

#### Description

Head wider than long. Mesoscutum large, posterior edge concave along midline; mesoscutellum triangular. Metatibia with one pair apical spurs, the spurs subequal to the diameter of the tibia. Metatarsi 5-segmented; tarsomere I ca. 3 times as long as tarsomere II; metatarsi with short spines.

Forewing ca. 2.8 times as long as wide; lacking humeral vein; prominent pterostigma present. Sc not forked. R_1_ forked distally; R_1b_ curved; Rs 4-branched; Rs_4_ to the apex of forewing; Rs_1+2_ stalked ca. 0.45 of vein total length; stem Rs_1+2_ as long as stem Rs_3+4_; s crossvein present. M 4-branched; M_1_, after separation from M_2_, subtends an angle of 63 (right wing) and 53 (left wing) degrees, sharply angulated at junction with r–m crossvein. Stem M_1+2_ shorter than stem M_3+4_; m crossvein originating near M_1+2_ furcation, ending at M_3+4_. CuA bifurcated; CuP simple. Crossvein m_3+4_–cua_1_ originating at ca. 1/3 length of M_3+4_ from its base and terminating near CuA furcation; cua–cup crossvein originating at ca. 2/3 length of CuA from its base and terminating slightly beyond midpoint of CuP. Anal veins looping into a double-Y configuration; distal part of 2A curved upward. Hind wing ca. 2.6 times as long as wide. Sc not forked. R_1_ not forked, the distal part curved; Rs 4-branched; stem Rs_1+2_ longer than stem Rs_3+4_. M 4-branched; M_1_ and M_2_ stalked; furcation of M_1+2_ at same level with furcation of Rs_1+2_. CuA bifurcated; CuP simple. Crossvein m_3_–cua_1_ present.

#### Measurements (in mm)

Body length ca. 8.1; width 1.8. Forewing length 8.8; width ca. 3.1. Hind wing length ca. 7.7; width 3.0.

### *Pegolepidopteron* Zhang, Shih, Labandeira & Ren gen. nov

urn:lsid:zoobank.org:act:EF716898-92B6-450F-BDC6-82E71602324E.

#### Type species

*Pegolepidopteron latiala* Zhang, Shih, Labandeira & Ren sp. nov

#### Etymology

This generic name is derived from the Greek *pegos* (strong, solid), in reference to the large body of this species, and *lepidos*, for “scale” or “flake,” also referring to this species’ assignment to the Lepidoptera; and *pteron*, for “wing” or “fin.” The gender is feminine.

#### Distribution

Inner Mongolia Autonomous Region, China.

#### Diagnosis

Body relatively large. Forewing with Sc forked, R_1_ forked. Crossvein r absent, r–m crossvein weak. Stem M_1+2_ longer than stem M_3+4_; m_3+4_–cua crossvein originating near M furcation and terminating at midpoint of CuA. Crossvein cua–cup located at the base of forewing. The distal part of 2A normal, not curved upward. Hind wing R_1_ forked.

An Ascololepidopterigidae affiliation is supported by: 1), antennae less than half the length of forewing; 2), wings homoneurous; 3), R_1_ forked; 4), M 4-branched; and 5), crossveins m–cua and cua–cup present.

#### Comparison

*Pegolepidopteron* differs from *Ascololepidopterix* in the following characters: 1), the forewing Sc is forked (vs. Sc not forked); 2), the r crossvein is present (vs. the r crossvein absent); 3), the m crossveins are absent (vs. the m crossveins present); 4), 2A is rectilinear (vs. the distal part of 2A curved upward); and 5), the hind wing R_1_ is forked (vs. R_1_ not forked and curved). When compared to *Agathiphaga* of the monogeneric Agathiphagidae, *Pegolepidopteron* can be separated by the following characters: 1), the R_1_ is forked (vs. the R_1_ not forked); 2), the sc–r crossvein is absent (vs. the sc–r crossvein present); 3), the r crossvein is present (vs. the r crossvein absent); 4), the location of the m_3+4_–cua crossvein and the cup–a crossvein; and 5), the hind wing venation of *Pegolepidopteron* is much different from that of *Agathiphaga*.

### *Pegolepidopteron latiala* Zhang, Shih, Labandeira & Ren sp. nov. (Fig. 19)

urn:lsid:zoobank.org:act:C34FB273-675E-4A4E-BA00-1589E5F70CAD.

#### Etymology

The specific name is derived from the Latin of *latus* (broad, wide), and *ala*, (a bird’s wing), in reference to the broad wings of this species.

**Figure 19:**
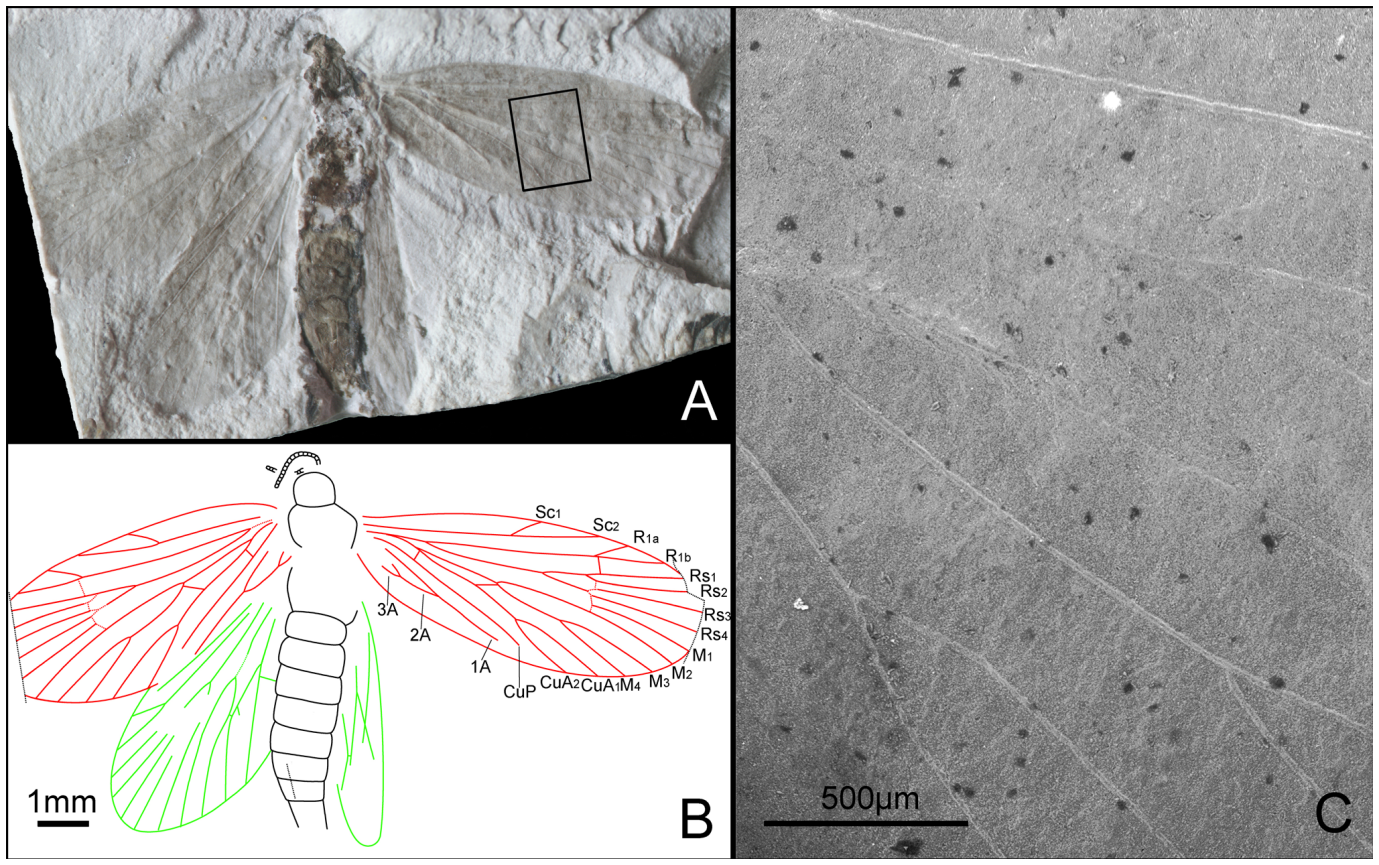
*Pegolepidopteron latiala* gen. et sp. nov. Female, holotype, CNU-LEP-NN-2012-001. (A), Fossil specimen. (B), Camera lucida drawing of (A), showing overall habitus. (C), SEM of right forewing venation, enlarged from the rectangular template in (A).

#### Type material

Holotype: CNU-LEP-NN-2012-001; ♀; well preserved forewings and hind wings.

#### Locality and horizon

This specimen was collected from Daohugou Village, Shantou Township, Ningcheng County, Inner Mongolia Autonomous Region, China. The age is latest Middle Jurassic, near the Callovian–Oxfordian boundary.

#### Diagnosis

Same as generic diagnosis.

#### Description

Antennae partially preserved, filiform, length of segments equal to their diameter.

Forewing ca. 2.5 times as long as wide; shape of wing apex unknown. Forewing lacking humeral vein. Sc forked, from distal 1/3 of vein stem; Sc_2_ extending to costal margin of wing at 2/3 length of wing from its base. Crossvein sc–r absent. R_1_ forked from distal 1/4 of stem, slightly beyond the Rs_1+2_ furcation; Rs 4-branched; Rs_1+2_ stalked ca. 0.45 of their vein length; stem Rs_1+2_ as long as stem Rs_3+4_; r crossvein located beyond the R_1_ furcation. M 4-branched; M_1_, after separation from M_2_, subtending an angle of 70 (left wing) and 100 (right wing) degrees, sharply angulated at junction with r–m crossvein; stem M_1+2_ slightly longer than stem M_3+4_; m_3+4_–cua crossvein slanted obliquely toward M_3+4_, originating near M furcation and terminating near the midpoint of CuA. Hyaline zones surrounding r–m crossveins at Rs_1+2_, Rs_3+4_ and M_1+2_ furcations. CuA bifurcated; CuP simple; cua–cup crossvein present, located at base of forewing. The stem of CuA diverges from stem of R basally, The stem of M diverges from stem of CuA beyond R-CuA furcation. Three anal veins loop into a double-Y configuration. Hind wing R_1_ forked; Rs 4-branched. M 4-branched. Crossvein m_3_–cua_1_ present between M_3_ and CuA; cua_2_-cup crossvein present. Anal area poorly preserved.

The anterior apophyses extending forward to distal margin of segment VII.

#### Measurements (in mm)

Body length ca. 7.7; width 1.2. Forewing length 7.2; width 2.9. Hind wing length ca. 5.8; width 2.8.

### *Trionolepidopteron* Zhang, Shih, Labandeira & Ren gen. nov

urn:lsid:zoobank.org:act:B2E35225-7FFD-4E66-B153-D6D638B6056E.

#### Type species

*Trionolepidopteron admarginis* Zhang, Shih, Labandeira & Ren sp. nov.

#### Etymology

The generic name is derived from the Greek *trion* (three) for the threefold branched Rs vein in the hind wing; and *lepidos* (scale, flake), referring to the Lepidoptera, the order to which this taxon is assigned; and *pteron* (a bird’s wing, fin).The gender is masculine.

#### Distribution

Inner Mongolia Autonomous Region, China.

#### Diagnosis

Body small; forewing with Sc forked. R_1_ forked. Crossvein rs_1_–rs_2_ present. Stem M_1+2_ longer than stem M_3+4_. Crossvein m_3+4_–cua located at the CuA furcation. Hind wing with both Rs and M 3-branched.

An Ascololepidopterigidae affiliation is supported by: 1), antennae less than half the length of forewing; 2), R_1_ forked; 3), M 4-branched; and 4), crossvein m–cua present.

#### Comparison

*Trionolepidopteron* resembles *Pegolepidopteron* in the venation of the forewing, including: 1), presence of the Sc and R_1_ forked veins; 2), the M vein is 4-branched; (3), a free M_1_ vein; and 4), presence of the m_3+4_–cua crossvein. *Trionolepidopteron* is differs from *Pegolepidopteron* by the following characters: 1), rs_1_–rs_2_ crossveins are present on the forewing (vs. absent); 2), r crossvein is absent on forewing (vs. present); 3), m_3+4_–cua crossvein is located near the CuA furcation (vs. more remote from the CuA furcation); and 4), the hind wing has a 3-branched Rs (vs. 4-branched). Differences also occur in gross body size, with *Trionolepidopteron* having a body length of ca. 4.0 mm long (vs. body lengths of more than 7.0 mm long for *Ascololepidopterix* and *Pegolepidopteron*).

### *Trionolepidopteron admarginis* Zhang, Shih, Labandeira & Ren sp. nov. (Fig. 20)

urn:lsid:zoobank.org:act:EC28A0BA-58AD-4630-860E-C558307500C1.

#### Etymology

The specific name is derived from the Latin preposition *ad* (toward, adjacent to), and *marginis* (border, edge), in reference to the rs_1_–rs_2_ crossvein occurring near the costal margin of the forewing.

**Figure 20:**
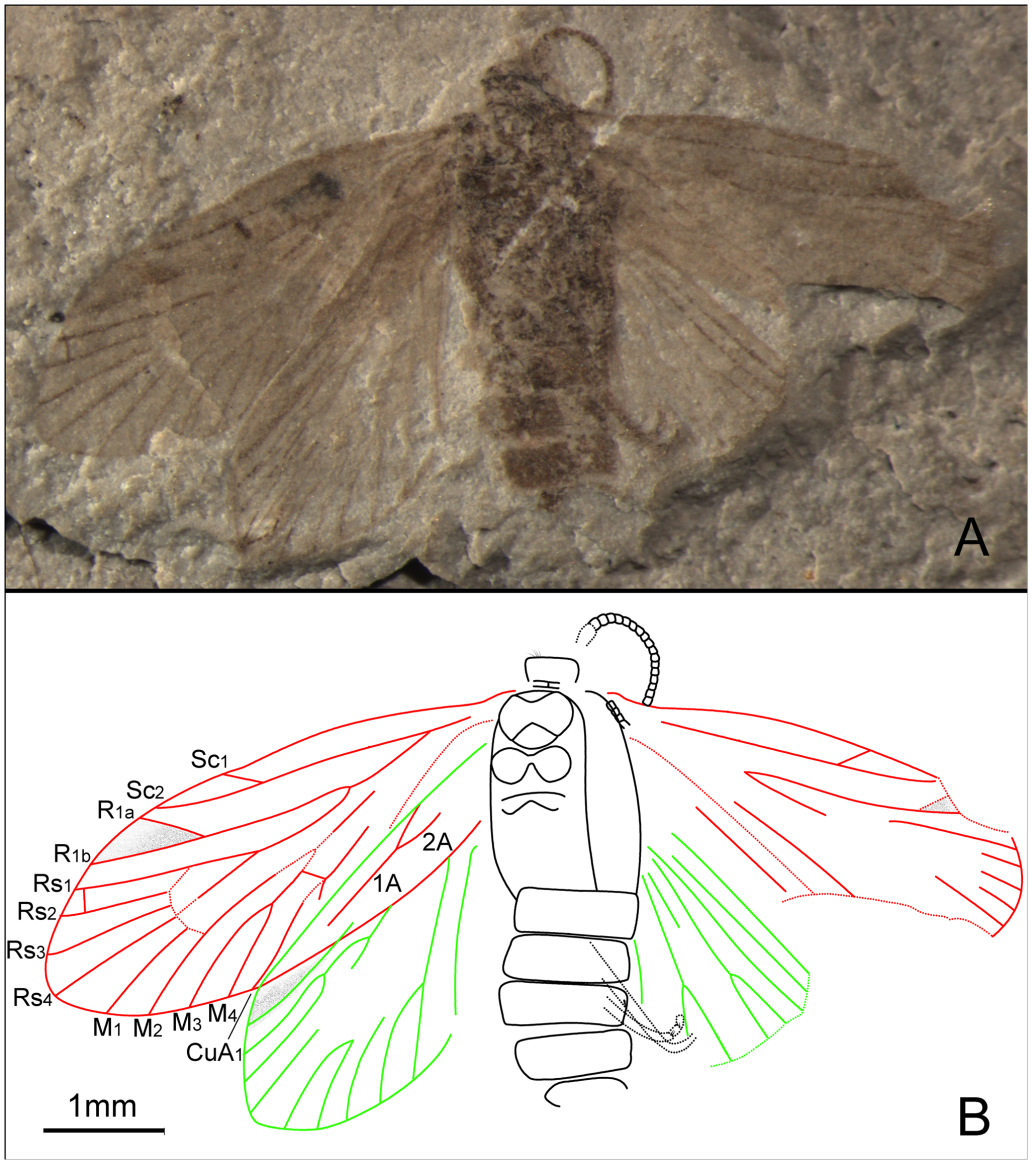
*Trionolepidopteron admarginis* gen. et sp. nov. Holotype, CNU-LEP-NN-2012-012. (A), Fossil specimen. (B), Camera lucida drawing of (A), showing overall habitus.

#### Type material

Holotype: CNU-LEP-NN-2012-012; sex unknown left forewing well preserved; anal area overlapping with hind wing.

#### Locality and horizon

This specimen was collected from Daohugou Village, Shantou Township, Ningcheng County, Inner Mongolia Autonomous Region, China. The age is latest Middle Jurassic, near the Callovian–Oxfordian boundary.

#### Diagnosis

Same as generic diagnosis.

#### Description

Antennae filiform; scape swollen; flagellum filiform; length of segments equal to their diameter. Short setae on anterior head margin. Mesothorax with small prescutum; mesoscutum broader than long; anterior and posterior edges of mesoscutum concave along midline; mesoscutellum fan-shaped, relatively large; mesoscutellum almost subequal to mesoscutum in length. Mesothorax slightly longer than metathorax; metascutum dumbbell shaped. Two short spines present on the apex of each tarsomere.

Forewing ca. 2.8 times as long as wide; pterostigma present. Humeral vein absent. Sc forked from 1/4 of vein stem distance; Sc_2_ extending to costal margin of wing, beyond 2/3 length of wing from base. R_1_ forked; sc–r crossvein absent; Rs 4-branched; Rs_4_ terminating at forewing apex; Rs_1+2_ stalked ca. 0.37 of their total length; stem Rs_1+2_ subequal to stem Rs_3+4_. Crossvein rs_1_–rs_2_ present and located near the costal margin of forewing. M 4-branched; stem M_1+2_ longer than stem M_3+4_; M_1_, after separation from M_2_ at an angle of ca. 70 degrees, sharply angulated at junction with r–m crossvein. Hyaline zones surround r–m crossveins at Rs_1+2_, Rs_3+4_, M_1+2_, and M_1+2_–M_3_ furcations. CuA bifurcated. Crossvein m_3+4_–cua present, originating on M_3+4_ and terminating near the CuA furcation. Hind wing ca. 2.5 times as long as wide. Sc and R_1_ not forked. Rs and M 3-branched. CuA bifurcate.

#### Measurements (in mm)

Body length over 3.8 as preserved (terminalia missing); width 1.6. Forewing length ca. 4.5; width ca. 1.6. Hind wing length ca. 3.7; width ca. 1.5.

### Family *Incertae sedis*

### Gen. et sp. *incertae sedis* (Fig. 21)

#### Material

CNU-LEP-NN-2012-013; ♂; antennae and forelegs well preserved; venation on both fore- and hind wings difficult to discern.

#### Locality and horizon

This specimen was collected from Daohugou Village, Shantou Township, Ningcheng County, Inner Mongolia Autonomous Region, China. The age is latest Middle Jurassic, near the Callovian–Oxfordian boundary.

#### Description

**Figure 21:**
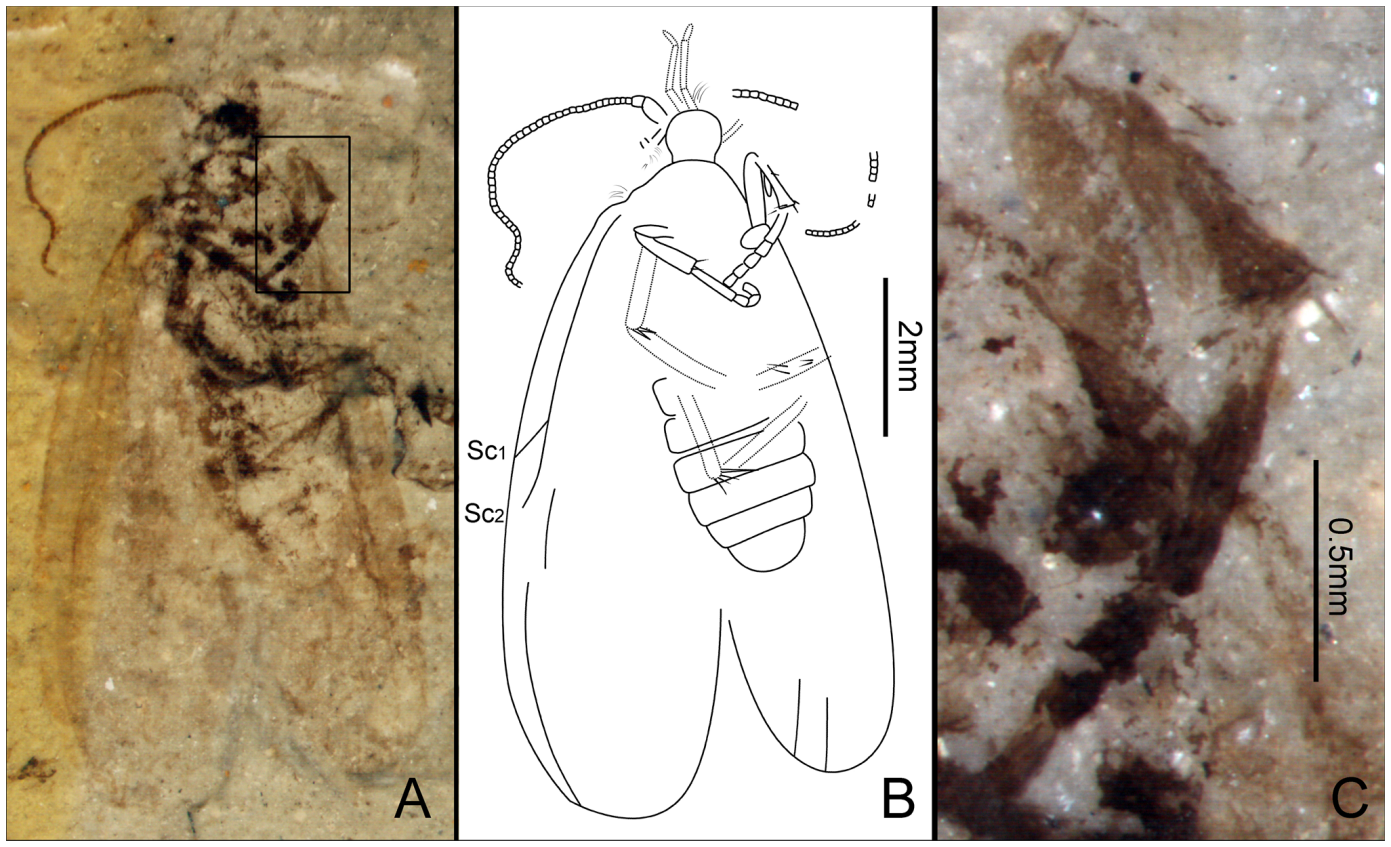
An unidentified moth. Male, CNU-LEP-NN-2012-013. (A), Fossil specimen. (B), Camera lucida drawing of (A), showing overall habitus. (C), Foreleg showing the tibial epiphysis, as outlined at the rectangular template in (A).

Antennae longer than half the length of forewing; scape swollen; pedicel relatively robust; flagellum filiform, length of flagellar segments equal to their diameter. Maxillary palpus long, the last segment short. Foretibia with epiphysis and spines (Fig. 21C); pretarsi 5-segmented. Both mesotibia and metatibia with one pair of apical spurs; medial spurs not discernible.

Forewings extremely long, distinctly exceeding end of the abdomen. Sc forked; other wing venation not discernible.

#### Measurements (in mm)

Body length 5.9; width 1.8. Forewing length 7.3; width ca. 2.6.

#### Remarks

The tibial epiphysis is well preserved on this specimen. The foretibia is characterized by well-developed epiphyses, an autapomorphy of the Lepidoptera. Based on this character, we consider this specimen is assigned to the Lepidoptera. However, due to indistinct venational characters, it cannot be placed in any lepidopteran family.

## Discussion

### Lepidopteran Characters from the Daohugou Specimens

Amphiesmenopteran fossils can be recognized by either the anal veins in the forewing forming a “double-Y” loop or by the hind wing M veins with at most three branches. Amphiesmenopteran specimens from the Middle Jurassic Jiulongshan Formation at Daohugou show that they had already acquired at least some lepidopteran or trichopteran apomorphies. Definitive Trichoptera and Lepidoptera from Daohugou previously have been reported by Gao *et al*. [43] and Huang *et al*. [25]. Daohugou Lepidoptera described here reveal an earlier underestimated diversity of Lepidoptera from this deposit. These specimens possess some of the most recently proposed lepidopteran autapomorphies.

Although Kristensen listed 23 autapomorphies for adult Lepidoptera [44], few of these can be observed on compression fossils. Grimaldi [8] specified three critical, external apomorphies that should be present in adults of early lepidopteran fossils: 1), absence of a forewing M_4_ vein; 2), presence of an epiphysis on the foretibia of (most) species; and 3), the occurrence of wing scales on both fore- and hind wings. In addition, the presence of mandibulate mouthparts [12], [40], [45], a well-developed jugum and associated setae [40], and a frenulum [12], [45] also are present and often used as diagnostic characters of basal moths [41].

One feature mentioned above, wing scales, have long been regarded as a diagnostic character for the Lepidoptera. Although a few trichopteran species also possess wing scales, they are always from rather derived lineages [46]. Grimaldi [8] mentioned that, differing from the Lepidoptera, the Trichoptera have wing scales that very occasionally occur on the forewings. However, some rare Trichoptera, such as *Monocentra lepidoptera* Rambur, do have wing scales on both wings [47], [48]. In primitive Lepidoptera, the scales are typically “solid”, lacking a lumen; wing-surface scales are non-perforate; and the outer surface of wing scales are densely set with transverse flutes [49]. Interestingly, only one lepidopteran fossil (CNU-LEP-NN-2012-024) from Daohugou exhibits evidence of scales, as robust piliform scales. Huang *et al*. [25] indicated the presence of wing scales from the Mesokristenseniidae, but their interpretation of the wing scales appears ambiguous. We believe that the absence of wing scales from Daohugou Lepidoptera is due to taphonomic factors. While scales are often preserved in amber ( [11], [12], and other examples), scales are known but seldom preserved in compression fossils. Another possible explanation involves the unlikely secondary loss of wing scales from Daohugou Lepidoptera. Such an explanation, however, would require an unparsimonious assumption that these lepidopterans represent a monophyletic lineage. The morphological diversity displayed in these specimens appears to undermine such an interpretation.

Based on our survey of the literature, we consider that the following four amphiesmenopteran apomorphies [15], [25] are sufficient for identifying specimens in this report as Amphiesmenoptera (Table 3).

**Table 3:** Amphiesmenopteran and Lepidopteran apomorphies for our specimens.

Genus	Species	Specimen	Amphiesmenopteran apomorphies^1^				Lepidopteran apomorphies^1^								
			1	2	3	4	5	6	7	8	9	10	11	12	13
*Sereslepidopteron*	*S. dualis*	CNU-LEP-NN-2006-001	O	O	?	?	?	O	?	O	?	O	O	?	?
		CNU-LEP-NN-2006-002	?	?	?	O	?	?	?	?	?	O	?	?	?
*Akainalepidopteron*	*A. elachipteron*	CNU-LEP-NN-2012-023	?	?	?	?	?	?	?	O	?	O	O	?	?
		CNU-LEP-NN-2012-024	?	O	?	?	?	?	?	O	?	O	O	?	?
		CNU-LEP-NN-2012-026	?	?	?	?	?	?	?	?	?	O	O	?	?
*Dynamilepidopteron*	*D. aspinosus*	CNU-LEP-NN-2012-014	?	?	?	?	?	?	?	O	?	O	?	?	?
*Quadruplecivena*	*Q. celsa*	CNU-LEP-NN-2012-027	?	O	?	?	?	?	O	O	?	O	X	?	?
*Petilicorpus*	*P. cristatus*	CNU-LEP-NN-2012-007	?	O	?	O	O	?	?	O	?	O	O	?	?
*Longcapitalis*	*L. excelsus*	CNU-LEP-NN-2012-025	O	O	?	?	?	?	?	?	?	O	O	?	?
*Grammikolepidopteron*	*G. extensus*	CNU-LEP-NN-2012-006	O	?	?	O	?	?	?	?	?	O	X	?	?
*Mesokristensenia*	*M. sinica*	CNU-LEP-NN-2009-003	?	?	?	?	?	?	?	O	?	X	O	?	?
	*M. trichophora*	CNU-LEP-NN-2012-032	O	?	?	?	?	?	?	O	?	X	O	?	?
*Kladolepidopteron*	*K. oviformis*	CNU-LEP-NN-2009-007	O	?	?	?	?	?	?	O	?	X	O	?	?
	*K. subaequalis*	CNU-LEP-NN-2012-020	O	O	?	?	?	?	?	?	?	X	O	?	?
		CNU-LEP-NN-2012-022	O	?	?	?	?	?	?	?	?	X	O	?	?
	*K. parva*	CNU-LEP-NN-2012-011	?	O	?	O	?	?	?	?	?	X	O	?	?
*Ascololepidopterix*	*A. multinerve*	CNU-LEP-NN-2012-028	O	O	?	?	?	?	?	?	?	X	O	?	?
*Pegolepidopteron*	*P. latiala*	CNU-LEP-NN-2012-001	O	O	?	O	?	?	?	?	?	X	O	?	?
*Trionolepidopteron*	*T. admarginis*	CNU-LEP-NN-2012-012	?	O	?	?	?	?	?	?	?	X	O	?	?
incertae sedis	?	CNU-LEP-NN-2012-013	?	?	?	?	?	O	O	O	?	?	?	?	?

1 Numbers refer to apomorphies listed in the text; O = character presence, X = character absence, ? = unknown state.

1. **The forewing anal veins form a “double-Y” loop**.
2. **The hind wing M vein bears at most three branches**. This condition also indicates that veins M_3_ and M_4_ are entirely merged.
3. **The pronotum has paired setose prominences**.
4. **The anterior margin of female segments VIII and IX are accompanied by long, rod-like apodemes**.
5. In addition, we cite the following, additional nine lepidopteran apomorphies [15], [25] as providing evidence for an affiliation to the Lepidoptera (Table 3).
6. **The median ocellus is absent**. The loss of median ocellus represents an autapomorphy of Lepidoptera, as this structure is present in Trichoptera [46]. The detailed structure of the head in most of these twenty specimens is difficult to discern. However, one of our better-preserved specimens, *Petilicorpus cristatus* gen. et sp. nov. (CNU-LEP-NN-2012-007, Fig. 10), displayed a head capsule in which a median ocellus was clearly lacking, indicating an assignment to the Lepidoptera.
7. **The foretibia possesses an epiphysis**. Except for the fossil genus *Mesokristensenia*, the epiphysis is a long-established and diagnostic character of modern Lepidoptera [46]. The foretibia bears a well-developed epiphysis and lacks apical spurs, often considered is an autapomorphous condition of the Lepidoptera. *Eolepidopterix jurassica*, belonging to the extinct lepidopteran family Eolepidopterigidae, possesses a foretibial epiphysis [4], [6]. In addition, an epiphysis occurs on the foretibia of *Seresilepidopteron dualis* gen. et sp. nov. (CNU-LEP-NN-2006-001, Fig. 3), and on specimen CNU-LEP-NN-2012-013, the latter a species of uncertain familial status (Fig. 21), confirming assignment of these two specimens to the Lepidoptera.
8. **The foretibia bears at most a single apical spur**. Except for the Agathiphagidae and Mesokristenseniidae that possess a single apical spur on their foretibiae, most other Lepidoptera lack foretibial spurs. A spur is absent on the foretibia of *Quadruplecivena celsa* gen. et sp. nov. (CNU-LEP-NN-2012-027, Figs, 8, 9), and on the unaffiliated species (CNU-LEP-NN-2012-013, Fig. 21), consistent with assignment of both specimens to the Lepidoptera.
9. **The mesotibia lacks medial spurs**. The absence of medial spurs on the tibiae of the Aglossata is consistent with an affiliation of some Daohugou specimens to the Lepidoptera.
10. **Absence of nygmata on the wings**. Nygmata are distinctive pustulate structures located at the base of the Rs_3_–Rs_4_ vein in almost all Trichoptera (Fig. 2B) [36]. By contrast, nygmata are absent in the Lepidoptera. All twenty specimens, except for one specimen with indeterminate wing venation, have wings that lack nygmata. The absence of nygmata on 19 specimens that exhibit good wing preservation cannot exclude the possible presence of nygmata, but strongly suggests that early Lepidoptera lacked these interesting wing structures.
11. **Forewing M vein is 3-branched**. A 3-branched medial vein, M, has long been considered a lepidopteran autapomorphy [6], but exceptions occur in both Lepidoptera and Trichoptera. For example, the M_4_ is present in the Aglossata, Mesokristenseniidae and Ascololepidopterigidae fam. nov. (Lepidoptera); and the M_4_ is absent in the extinct family Dysoneuridae (Trichoptera). In spite of these exceptions, most of the specimens with 3-branched M veins (Figs. 1C, D, E and 2A) are most likely Lepidoptera.
12. **Separation of the M_1_ from the M_2_ veins is accompanied by an angle greater than 60 degrees and a sharp, angulate intersection at the junction with the r-m crossvein** (Fig. 2A). Huang *et al*. [25] proposed that Lepidoptera can be distinguished from Trichoptera by this character of the M_1_ vein. In Lepidoptera, the M_1_ usually separates from M_2_ at an angle greater than 60 degrees, followed by sharp angulation at junction with the r–m crossvein (Figs. 1C, F, and 2A). By contrast, nearly all Trichoptera have branches of the M_1+2_ with the M_1_ and M_2_ veins at an angle of less than 45 degrees; in addition, and M_1_ is linear and smooth (Figs. 1G and 2B). Not all Lepidoptera have a M_1_ vein that is sharply angulated at the junction with r–m crossvein, such as *Undopterix sukatshevae* that was identified as a lepidopteran based on the presence of scales (Fig. 1D), and *Netoxena nana* based on its 3-branched M vein (Fig. 1E). If a fossil species has this feature, it is most likely a lepidopteran. Most of our twenty specimens exhibit wings with this character, except for two lacking this feature and three that have it as an ambiguous presence (as shown in Table 3).
13. **The male valva is primarily undivided**.
14. **The cerci are absent in both sexes**.

### The Early Evolution of the Amphiesmenoptera

The Amphiesmenoptera likely originated from the stem panorpoid stock [15]. Although no fossils unambiguously represent the common ancestry of Lepidoptera and Trichoptera, the extinct family Necrotauliidae has been suggested as the best candidate (Fig. 1A) [5]. This family is a paraphyletic assemblage of species that existed from the Triassic to the Cretaceous [50]. The earliest fossil member of the Lepidoptera, *Archaeolepis mane* (Fig. 1B), was reported from Sinemurian Stage, of the mid Early Jurassic age, an assignment based primarily on distinctive lepidopteran scales [18]. Characters of the wing venation, legs and axial part of the body of the various new species in this report indicate the presence of autapomorphies in both the Amphiesmenoptera and the Lepidoptera. These features provide supplemental evidence to support the hypothesized divergence of the Lepidoptera and Trichoptera occurring by the Early Jurassic. This differentiation of the Lepidoptera and Trichoptera likely had transpired during the Triassic–Jurassic boundary [50], or possibly Late Triassic [6], [51]. Notably, gracile, lepidopteran-like, epidermal leaf mines reminiscent of the Nepticulidae and more basal monotrysian leaf mining lineages are present in a Late Triassic (Carnian) flora from South Africa (C Labandeira, R Prevec, E Currano, J Anderson and H Anderson, unpublished data), perhaps indicating an earlier, Middle Triassic date for the origin of basalmost lepidopteran lineages.
